# Diagnostic testing and the evolution of detection avoidance by pathogens

**DOI:** 10.1093/emph/eoae018

**Published:** 2024-08-27

**Authors:** Jason Wood, Ben Ashby

**Affiliations:** Department of Mathematical Sciences, University of Bath, Bath, UK; Department of Mathematical Sciences, University of Bath, Bath, UK; Department of Mathematics, Simon Fraser University, Vancouver, Canada; The Pacific Institute on Pathogens, Pandemics and Society (PIPPS), Simon Fraser University, Burnaby, BC, Canada

**Keywords:** disease surveillance, evolution, diagnostic test escape, public health

## Abstract

Diagnostic testing is a key tool in the fight against many infectious diseases. The emergence of pathogen variants that are able to avoid detection by diagnostic testing therefore represents a key challenge for public health. In recent years, variants for multiple pathogens have emerged which escape diagnostic testing, including mutations in *Plasmodium falciparum* (malaria), *Chlamydia trachomatis* (chlamydia) and SARS-Cov-2 (Severe acute respiratory syndrome coronavirus 2) (Coronavirus disease 2019). However, little is currently known about when and the extent to which diagnostic test escape will evolve. Here we use a mathematical model to explore how the frequency of diagnostic testing, combined with variation in compliance and efficacy of isolating, together drive the evolution of detection avoidance. We derive key thresholds under which a testing regime will (i) select for diagnostic test avoidance, or (ii) drive the pathogen extinct. Crucially, we show that imperfect compliance with diagnostic testing regimes can have marked effects on selection for detection avoidance, and consequently, for disease control. Yet somewhat counterintuitively, we find that an intermediate level of testing can select for the highest level of detection avoidance. Our results, combined with evidence from various pathogens, demonstrate that the evolution of diagnostic testing avoidance should be carefully considered when designing diagnostic testing regimes.

## INTRODUCTION

Pathogens may evolve in response to human interventions, which presents a key challenge for public health policymakers. For example, pharmaceutical interventions such as antimicrobials or vaccines, can lead to the evolution of antimicrobial resistance (AMR) [1] or vaccine escape [2], respectively. Pathogen evolution can therefore have dramatic effects on the efficacy of public health interventions by hindering efforts to treat or prevent infections. Consequently, substantial efforts have been devoted to understanding how pathogens evolve in response to public health policies, especially pharmaceutical interventions, and devising optimal strategies to mitigate or constrain pathogen evolution [3].

While much attention has been paid to the impact of pharmaceutical interventions on pathogen evolution, non-pharmaceutical interventions (NPIs), such as diagnostic testing [4, 5] or reducing social mixing [6] may also affect pathogen evolution. For example, routine surveillance of *Clostridium difficile* in Costa Rica identified three variants which gave negative PCR test results, caused by deletion of the *tcdC* gene [7]. Similarly, in SARS-Cov-2 (Severe accute respiratory syndrome coronavirus 2), a deletion within the N gene can produce false-negative antigen tests [8, 9], and in *Plasmodium falciparum*, variants lacking the histidine-rich protein 2 (pfhrp2) and 3 (pfhrp3) genes have been identified following the introduction of rapid diagnostic tests targeting these antigens [5]. Similar patterns have been observed in STIs. In *Chlamydia trachomatis,* a variant with a 377 bp deletion has previously escaped diagnostic PCR testing [4, 10], and in *Neisseria gonorrhoeae* it has been shown that diagnostic escape could occur through a reversion in the *gyrA* allele (the gene encoding the A subunit of DNA gyrase) [11]. Pathogen evolution arising from NPIs therefore has the potential for significant adverse effects on public health responses for many pathogens, akin to the effects of pharmaceutical interventions. Detection avoidance arising from diagnostic testing is of particular concern. Without reliable diagnostics, patients may experience prolonged symptoms or unknowingly transmit the disease. Understanding precisely how diagnostic testing affects pathogen evolution is crucial for assessing the risk of inducing selection for detection avoidance, and its impact on population health.

Theoretical studies on the evolution of detection avoidance have primarily focussed on selection for asymptomatic infections [12, 13], and by comparison relatively little is known about escape from diagnostic testing. As far as we are aware, only four studies have explored the evolution of detection avoidance arising from diagnostic testing. Watson et al. [14] explored a mathematical model for the spread of a variant of *P. Falciparum* (the causative agent of malaria) which has been observed to escape Rapid Diagnostic Test kits (RDTs) due to gene deletions in histidine-rich protein 2 (HRP2). They found that selection for detection avoidance was most prevalent when malaria transmission was low, and patients were seeking and receiving treatment frequently based on diagnosis with RDTs. Smid [15] used a spatially structured mathematical model of chlamydia transmission in Sweden to explore the emergence of a *C. trachomatis* variant known to escpe detection by two diagnostic tests due to a plasmid deletion. They found that the *C. trachomatis* variant spread more favourably in counties which used both nucleic acid amplification tests, but once the testing regime had been updated to detect the new variant, the proportion of the population affected by the variant dramatically decreased. This could indicate a likely evolutionary trade-off associated with detection avoidance, which has also been speculated for the deletion of HRP2 in *P. falciparum* [14]. Similarly, Del Vecchio et al. [9] explored antigen test target failure in primary care settings in Italy for SARS-Cov-2. They found that a variant which produced false-negative antigen tests was selected for in the region of Veneto, in which a higher proportion of tests were antigen rather than PCR (Polymerase Chain Reaction). Del Vecchio et al. [9] also found that the variant which produced false-negative tests likely had a lower reproduction number than the Alpha variant, which was dominant at the time. Gurevich et al. [16] explored the evolution of SARS-Cov-2 using a two strain eco-evolutionary model. They found that as the test-evasive strain became more detectable, testing rates needed to increase to select for the evasive strain, however as the strength of NPIs increased the required testing rate decreased.

Here, we investigate how different testing regimes and the frequency or probability of taking a test affects selection for diagnostic test escape. In particular, we focus on how the effectiveness of (and compliance with) subsequent public health measures to reduce transmission (i.e. isolation) mediates detection avoidance evolution. We derive key thresholds for (i) pathogen extinction due to testing, and (ii) detection avoidance to evolve, and show how evolution can prevent extinction. Crucially, we also show that an intermediate rate of testing can lead to the highest level of detection avoidance.

## METHODS

### Model description

We consider the epidemiological and evolutionary dynamics of a directly transmitted pathogen in a well-mixed population of hosts, where S is the density of healthy (uninfected) hosts, Q and A are the densities of hosts who are aware of their infection and are either isolating or not isolating, respectively, U  is the density of hosts who are not aware that they are infected, and R is the density of hosts who have recovered. The total density of the population is then N=S+Q+A+U+R. Hosts reproduce at a baseline rate b, which is not affected by infection status, but is subject to a density-dependent crowding affect, qN, where q>0. Pathogen transmission is a function of detectability, ρ, and is density dependent at baseline rate β(ρ), with force of infection, λ(ρ)=β(ρ)(δQ+A+U), where δ∈[0,1] is the reduction in transmission due to isolation. We assume that there is no link between awareness of the infection and the rate of recovery, γ, or disease-associated mortality (virulence), α, and that hosts who are not aware of their infection do not change their behaviour. A full description of the model parameters can be found in Table 1.

**Table 1.** Parameter/ variable descriptions and default values

Parameter/ variable	Description	Default value(s)
b	Host birth rate	2.0
$c_{1}$	Strength of trade-off	1.0
$c_{2}$	Curvature of trade-off	−3.0
d	Host natural death rate	0.1
m	Probability of new infection being a mutant	0.2
q	Strength of density-dependent competition	0.1
α	Disease-associated mortality rate (virulence)	0.5
$\beta_{0}$	Maximum pathogen transmission rate	1.0
γ	Host recovery rate	0.5
δ	Effectiveness of isolating measures	0,0.1
η	Probability of host compliance with isolating measures	1,0.9
λ	Force of infection	n/a
ρ	Probability of a false-negative test	n/a
σ	Probability of taking a test in Model A	0.75
ζ	Population testing rate in Models B and C	3

We consider three versions of the model to account for different testing scenarios. In all three cases, ρ is the probability of a false-negative test, which we assume to be under selection in the pathogen (see below), and η is the probability that an individual complies with isolating measures. In the first version (Model A), all infected hosts take a single test with probability σ∈[0,1] when they initially become symptomatic, with the onset of symptoms coinciding with the beginning of the infectious period for all hosts. Upon infection, individuals with a positive test move into the isolating (Q) class with probability ση(1−ρ) and into the aware-but-not-isolating class (A) with probability σ(1−η)(1−ρ). Individuals with a false-negative test move into the unaware class (U) with probability 1−σ(1−ρ). The ecological dynamics for Model A are given by:


$$\begin{array}{l} \dot{S}=b\left(1-qN\right)N-\left(\lambda \left(\rho \right)+d\right)S \end{array}$$

$$\begin{array}{l} \;\dot{U}=\left(\sigma \rho +\left(1-\sigma \right)\right)\lambda \left(\rho \right)S-\left(d+\alpha +\gamma \right)U \end{array}$$

$$\begin{array}{l} \dot{Q}=\sigma \left(1-\rho \right)\eta \lambda \left(\rho \right)S-\left(d+\alpha +\gamma \right)Q \end{array}$$ (1)

$$\begin{array}{l} \dot{A}=\sigma \left(1-\rho \right)\left(1-\eta \right)\lambda \left(\rho \right)S-\left(d+\alpha +\gamma \right)A \end{array}$$

$$\begin{array}{l} \dot{R}=\gamma \left(Q+A+U\right)-dR \end{array}$$

In the second version (Model B), infected hosts initially enter the unaware class (U) and test regularly at rate ζ with a probability ρ of returning a false negative per test (the outcome of tests are therefore independent). Infected individuals move to the isolating class at rate ζ(1−ρ)η and to the aware-but-not-isolating class (A) at rate ζ(1−ρ)(1−η). The ecological dynamics for Model B are given by:


$$\begin{array}{l} \dot{S}=b\left(1-qN\right)N-\left(\lambda \left(\rho \right)+d\right)S \end{array}$$

$$\begin{array}{l} \dot{U}=\lambda \left(\rho \right)S-\zeta \left(1-\rho \right)U-\left(d+\alpha +\gamma \right)U \end{array}$$

$$\begin{array}{l} \dot{Q}=\zeta \left(1-\rho \right)\eta U-\left(d+\alpha +\gamma \right)Q \end{array}$$ (2)

$$\begin{array}{l} \dot{A}=\zeta \left(1-\rho \right)\left(1-\eta \right)U-\left(d+\alpha +\gamma \right)A \end{array}$$

$$\begin{array}{l} \dot{R}=\gamma \left(Q+A+U\right)-dR \end{array}$$

In the third version (Model C), hosts either enter a permanently unaware class (U) with probability (ρ) or the temporarily unaware class (I) with probability (1−ρ). Temporarily unaware hosts then move into the isolating class (Q) at a rate ζη and into the aware-but-not-isolating class (A) with rate ζ(1−η). As the testing rate gets very large (ζ→∞), Model C becomes equivalent to Model A. In Model C, N=S+U+I+A+Q+R, and λ(ρ)=β(ρ)(δQ+A+U+I) The ecological dynamics of Model C are given by:


$$\begin{array}{l} \dot{S}=b\left(1-qN\right)N-\left(\lambda \left(\rho \right)+d\right)S \end{array}$$

$$\begin{array}{l} \dot{U}=\rho \lambda \left(\rho \right)S-\left(d+\alpha +\gamma \right)U \end{array}$$

$$\begin{array}{l} \dot{I}=\left(1-\rho \right)\lambda \left(\rho \right)S-\zeta I-\left(d+\alpha +\gamma \right)I \end{array}$$ (3)

$$\begin{array}{l} \dot{Q}=\zeta \eta I-\left(d+\alpha +\gamma \right)Q \end{array}$$

$$\begin{array}{l} \dot{A}=\zeta \left(1-\eta \right)I-\left(d+\alpha +\gamma \right)A \end{array}$$

$$\begin{array}{l} \dot{R}=\gamma \left(Q+A+U+I\right)-dR \end{array}$$

For all versions of the model, we investigate how compliance (η) and the effectiveness of isolating (δ) affect selection for detection avoidance (ρ) in the pathogen. We assume there is a trade-off between pathogen transmission, β, and the likelihood of producing a false-negative test, ρ, such that β=β(ρ) and hence λ=λ(ρ). Mechanistically, we are assuming that the more transmission stages that are produced, the more likely these are to be detected by the test, and so an increase in transmission comes at the cost of being more detectable, while detection avoidance comes at the cost of a lower transmission rate, i.e. $\frac{\partial \beta }{\partial \rho }<0$. This assumption is motivated by data from chlamydia, SARS-Cov-2 and malaria infections, where variants that are less detectable by PCR and rapid testing also appear to be less transmissible [5, 10]. There is no a priori reasoning for the curvature of the trade-off, so we explore both accelerating and decelerating trade-offs of the form,


$$\begin{array}{l} \beta \left(\rho \right)=\beta_{0}\left(\left(1-c_{1}\right)+c_{1}\frac{1-e^{c_{2}\left(1-\rho \right)}}{1-e^{c_{2}}}\right) \end{array}$$ (4)

where $c_{1}$ controls the strength of the trade-off and $c_{2}\neq 0$ the curvature, with $c_{2}>0$ corresponding to accelerating costs of detection avoidance on the transmission rate (diminishing returns of detection avoidance) and $c_{2}<0$ corresponding to decelerating costs (increasing returns of detection avoidance).

We assume that mutations are sufficiently rare that the epidemiological dynamics (equations 1–3) reach a stable endemic equilibrium before a mutant arises and that mutations have small phenotypic effects. A rare new mutant pathogen with false negativity probability $\rho_{m}$ will then have invasion dynamics,


$${\dot{U}}_{m}=\left(\sigma \rho_{m}+\left(1-\sigma \right)\right)\lambda \left(\rho_{m}\right)S^{*}-\left(d+\alpha +\gamma \right)U_{m}$$

$${\dot{Q}}_{m}=\sigma \left(1-\rho_{m}\right)\eta \lambda \left(\rho_{m}\right)S^{*}-\left(d+\alpha +\gamma \right)Q_{m}$$ (5)

$${\dot{A}}_{m}=\sigma \left(1-\rho_{m}\right)\left(1-\eta \right)\lambda \left(\rho_{m}\right)S^{*}-\left(d+\alpha +\gamma \right)A_{m}$$

in Model A, and


$${\dot{U}}_{m}=\lambda \left(\rho_{m}\right)S^{*}-\zeta \left(1-\rho_{m}\right)U_{m}-\left(d+\alpha +\gamma \right)U_{m}$$

$${\dot{Q}}_{m}=\zeta \left(1-\rho_{m}\right)\eta U_{m}-\left(d+\alpha +\gamma \right)Q_{m}$$ (6)

$${\dot{A}}_{m}=\zeta \left(1-\rho_{m}\right)\left(1-\eta \right)U_{m}-\left(d+\alpha +\gamma \right)A_{m}$$

in Model B, and


$${\dot{U}}_{m}=\rho \lambda \left(\rho_{m}\right)S^{*}-\left(d+\alpha +\gamma \right)U_{m}$$

$${\dot{I}}_{m}=\left(1-\rho_{m}\right)\lambda \left(\rho_{m}\right)S^{*}-\zeta I_{m}-\left(d+\alpha +\gamma \right)I_{m}$$

$${\dot{Q}}_{m}=\zeta \eta I_{m}-\left(d+\alpha +\gamma \right)Q_{m}$$

$${\dot{A}}_{m}=\zeta \left(1-\eta \right)I_{m}-\left(d+\alpha +\gamma \right)A_{m}$$

in Model C, where asterisks denote the resident population at equilibrium.

We calculate the invasion fitness, $r\left(\rho_{m},\rho \right)$, of a rare mutant using the Next Generation Method [17], and subsequently the fitness gradient, $F\left(\rho \right)={\frac{\partial r\left(\rho_{m},\rho \right)}{\partial \rho_{m}}|}_{\rho_{m}=\rho }$ (see Supplementary Material), as:


$$\begin{array}{l} \;F\left(\rho \right)=\frac{-S^{*}\left(\left(-1+\eta \left(\delta -1\right)\left(\rho -1\right)\sigma \right)\beta^{\prime }\left(\rho \right)+\beta \left(\rho \right)\eta \sigma \left(\delta -1\right)\right)}{d+\alpha +\gamma } \end{array}$$ (8)

in Model A,


$$\begin{array}{llll} \;F(\rho )= & \;\frac{1}{(d+\alpha +\gamma ){\left(\zeta (1-\rho )+d+\alpha +\gamma \right)}^{2}}(((\zeta (1-\rho )+d+\alpha +\gamma )\; & \;(-(1+(\delta -1)\eta )(\rho -1)\zeta +d+\alpha +\gamma )\beta^{\prime }(\rho )\; & \;-\eta \zeta \beta (\rho )(\delta -1)(d+\alpha +\gamma ))S^{*}) \end{array}$$ (9)

in Model B, and


$$\begin{array}{lll} & F\left(\rho \right)\; & =-\frac{S\left(\beta^{\prime }\left(\rho \right)\left(\left(-1+\left(\rho -1\right)\left(\delta -1\right)\eta \right)\zeta -\left(d+\alpha +\gamma \right)\right)+\eta \zeta \beta \left(\rho \right)\left(\delta -1\right)\right)}{\left(d+\alpha +\gamma \right)\left(d+\alpha +\gamma +\zeta \right)} \end{array}$$ (10)

in Model C.

A singular strategy, $\rho^{*}$, is a value of ρ which satisfies $\;F\left(\rho^{*}\right)=0$. The evolutionary stability of a singular strategy is determined by the sign of $E\left(\rho^{*}\right)={\frac{\partial^{2}r\left(\rho_{m},\rho \right)}{\partial \rho_{m}^{2}}|}_{\rho_{m}=\rho =\rho^{*}}$, and the convergence stability is determined by the sign of $E\left(\rho^{*}\right)+M\left(\rho^{*}\right)\;$ where $M\left(\rho^{*}\right)={\frac{\partial^{2}r\left(\rho_{m},\rho \right)}{\partial \rho_{m}\partial \rho }|}_{\rho_{m}=\rho =\rho^{*}}$. A singular strategy, $\rho^{*}$, is evolutionary stable if $E\left(\rho^{*}\right)<0$, and convergence stable if $E\left(\rho^{*}\right)+M\left(\rho^{*}\right)<0$. In all three models, it can be shown that $M\left(\rho^{*}\right)={\frac{\partial^{2}r\left(\rho_{m},\rho \right)}{\partial \rho_{m}\partial \rho }|}_{\rho_{m}=\rho =\rho^{*}}=0$, hence meaning any evolutionarily stable point is also convergence stable, and branching is not possible (see Supplementary Material*).*

Adaptive dynamics focuses on long-term evolutionary dynamics. To explore short-term evolutionary dynamics during the expansion phase of an epidemic, we used stochastic simulations by implementing the Gillespie algorithm [18]. Specifically, we create a discretised trait space for ρ, and assume that each new infection has a probability (m=0.2) of mutating to the adjacent strain. We assume the population size is large but non-replenishing (N=100 000). We initialize simulations with 10 infected individuals and N−10 susceptible individuals. We normalize each simulation by the time taken to reach the epidemic peak to compare across testing rates, as the time taken to reach the epidemic peak increases with the testing rate. At each recording step, we calculate the average level of detection avoidance and track the number of infected hosts. For each set of parameter values, we run 20 simulations. We also compare our adaptive dynamics results to long-term stochastic simulations in a smaller, but replenishing population where any host that dies is replaced by a new susceptible, and recovered hosts re-enter the susceptible pool (N=1000). All code used to produce the figures within this article is available within the Supplementary material and on GitHub.

## RESULTS

### No compliance

Trivially, if there is no compliance with isolating measures (η=0), then the fitness gradient in all three models is


$$\begin{array}{l} F\left(\rho \right)=\frac{S^{*}\beta^{\prime }\left(\rho \right)}{d+\alpha +\gamma }<0 \end{array}$$ (11)

since $\beta^{\prime }\left(\rho \right)$ is negative for all ρ. The pathogen will therefore evolve to produce as few false-negative tests as possible (ρ→0), favouring a higher rate of transmission (the same outcome occurs if isolating has no effect on transmission (δ=1)).

### Perfect compliance and isolating

From the fitness gradients (equations 8–10), we can deduce that the strongest selection for false-negative tests (highest value of $\rho^{*}$) will occur when there is perfect compliance with isolating measures (η=1) and isolating completely prevents transmission (δ=0). In Model A, the singular strategy satisfies,


$$\begin{array}{l} \beta {\left(\rho^{*}\right)}^{\prime }=\frac{-\sigma \beta \left(\rho^{*}\right)}{\sigma \rho^{*}+\left(1-\sigma \right)} \end{array}$$ (12)

in Model B satisfies


$$\begin{array}{l} \beta {\left(\rho^{*}\right)}^{\prime }=\frac{-\zeta }{S^{*}}=\frac{-\zeta \beta \left(\rho^{*}\right)}{\zeta \left(1-\rho^{*}\right)+d+\alpha +\gamma } \end{array}$$ (13)

and in Model C satisfies


$$\begin{array}{l} \beta {\left(\rho^{*}\right)}^{\prime }=\frac{-\zeta \beta \left(\rho^{*}\right)}{\zeta \rho^{*}+d+\alpha +\gamma } \end{array}$$ (14)

In Model A, there is only a single opportunity to move into the ‘aware’ class as only one test is taken at the onset of symptoms, with probability σ. The probability of taking a test therefore plays a crucial role in driving selection for an intermediate probability of false positives, depending on the strength of the trade-off with transmission. In Model B, hosts test at a continuous rate leading to an additional flux out of the infectious class, similar to death (d+α) and recovery (γ). In Model C there is one opportunity to move into the aware classes, but the rate of testing varies. As a result, the optimal probability of false negativity depends on how the rate of testing (ζ) compares to the rate of movement out of the class due to other processes. When testing is maximal (σ=1 in Model A and ζ→∞ in Model B and Model C) any non-zero probability of false-negative tests (ρ>0) results in some individuals moving into the unaware infected class (U) in Model A and Model C, whereas in Model B the unaware class is only non-empty if the pathogen produces exclusively false-negative tests (ρ=1).

**Figure 1.**
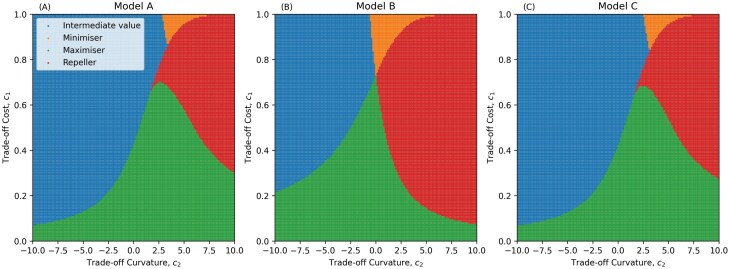
Evolutionary outcomes for Model A (A), Model B (B) and Model C (C), for different trade-off curvatures $\left(c_{2}\right)$ and trade-off costs $\left(c_{1}\right)$. The pathogen experiences accelerating costs (diminishing returns) of detection avoidance when $c_{2}<0$, and decelerating costs (increasing returns) when $c_{2}>0$. Blue regions indicate the pathogen evolves to an intermediate level of detection avoidance (0<ρ<1). The orange regions indicate where the pathogen does not evolve detection avoidance (ρ=0). In the green regions, the pathogen maximizes detection avoidance (ρ=1). In the red regions, there is an evolutionary repeller at an intermediate value of ρ, above which selection maximizes detection avoidance (ρ=1), and below which selection minimizes detection avoidance (ρ=0). Parameters as in Table 1 except where specified, δ=0, η=1. A colour version of this figure appears in the online version of this article.

**Figure 2.**
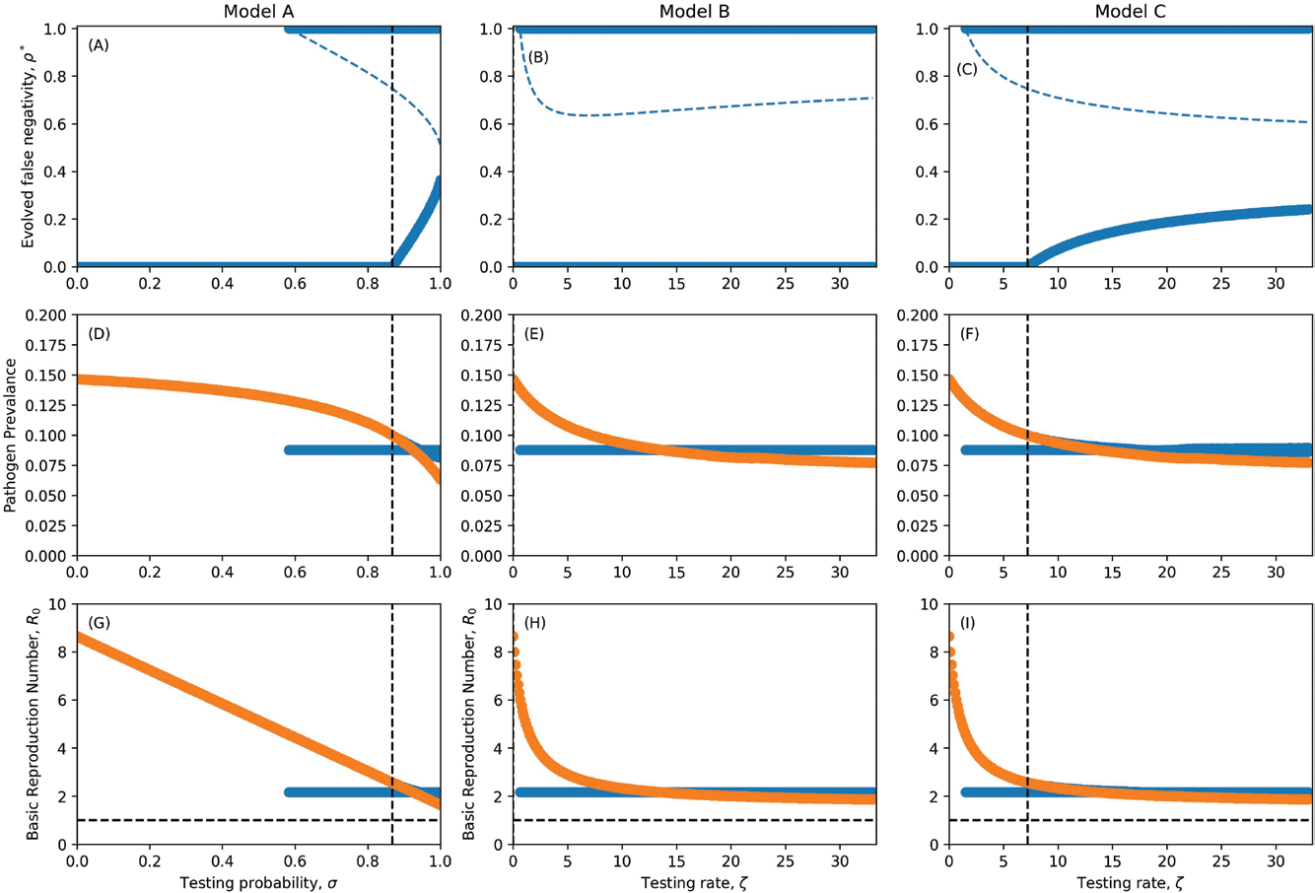
Evolved levels of false negativity with imperfect isolating (A–C), corresponding pathogen prevalence (D–F) and reproduction numbers (G–I). Dashed blue line (A–C) represents the evolutionary repellers. Dashed vertical line denotes level of testing required to select for false-negative tests. Dotted and Dashed line indicates extinction thresholds. In Reproduction number plots a horizontal dashed line indicates the value $R_{0}=1$. In pathogen prevalence and reproduction number plot, blue points indicate prevalence or $R_{0}$ of singular strategy, orange points indicate prevalence or $R_{0}$ in the absence of evolution. Parameters as in Table 1 except where specified, $c_{2}=3.0,\;\delta =0.1,\;\eta =0.9$. A colour version of this figure appears in the online version of this article.

If detection avoidance is increasingly costly to transmission $\left(c_{2}<0\right)$, the pathogen always evolves to an evolutionarily stable strategy, including the boundaries ρ=0 and ρ=1 (Fig. 1). This is very similar to the behaviour seen in many transmission-virulence trade-offs, where accelerating costs lead to intermediate levels of virulence evolution [19]. Accelerating costs imply diminishing returns, which generally favours an intermediate optimal level of detection avoidance. However, if detection avoidance is decreasingly costly $\left(c_{2}>0\right)$ the pathogen may evolve to either maximize (ρ=1) or minimize (ρ=0) detection avoidance depending on the initial conditions due to the presence of an evolutionary repeller. This is because decelerating costs mean there is a disproportionately high cost of false negativity when ρ is relatively small, which tends to select against detection avoidance, whereas there is a disproportionately low cost when ρ is sufficiently high, which tends to select for greater detection avoidance (i.e. an evolutionary repeller). The existence of an evolutionary repeller in the decelerating cost space means that the outcome typically depends on the initial level of detection avoidance (Fig. 2). In Model B, for example, the only possible outcomes are no or complete detection avoidance (Fig. 2B). For Models A and C, higher testing probabilities/rates increase the lower bound on evolved false negativity, $\rho^{*}$ and increase the basin of attraction (above the repeller) for complete detection avoidance (Fig. 2A, C). Hereafter we will assume detection avoidance is I ncreasingly costly $\left(c_{2}<0\right)$ as this is the only scenario where an intermediate level of false negativity may evolve in all three models.

As testing increases, one might naively have expected the optimal level of false-negative tests to also increase (in all of the models). However, if we imagine low testing, e.g. σ≈0 or ζ≈0, we would not expect to see selection for detection avoidance as there is a cost to transmission of producing false-negative tests. From this, we can infer that there is a threshold wherein testing needs to be sufficiently common before selection favours detection avoidance. In the Supplementary Material we derive the minimum test probability (Model A) and minimum testing rate (Models B and C) needed for detection avoidance to evolve. Similarly, if testing is sufficiently common (large σ or ζ), isolating is sufficiently effective (large δ) and compliance is sufficiently high (large η), it may be possible for the pathogen to be driven extinct.

**Figure 3.**
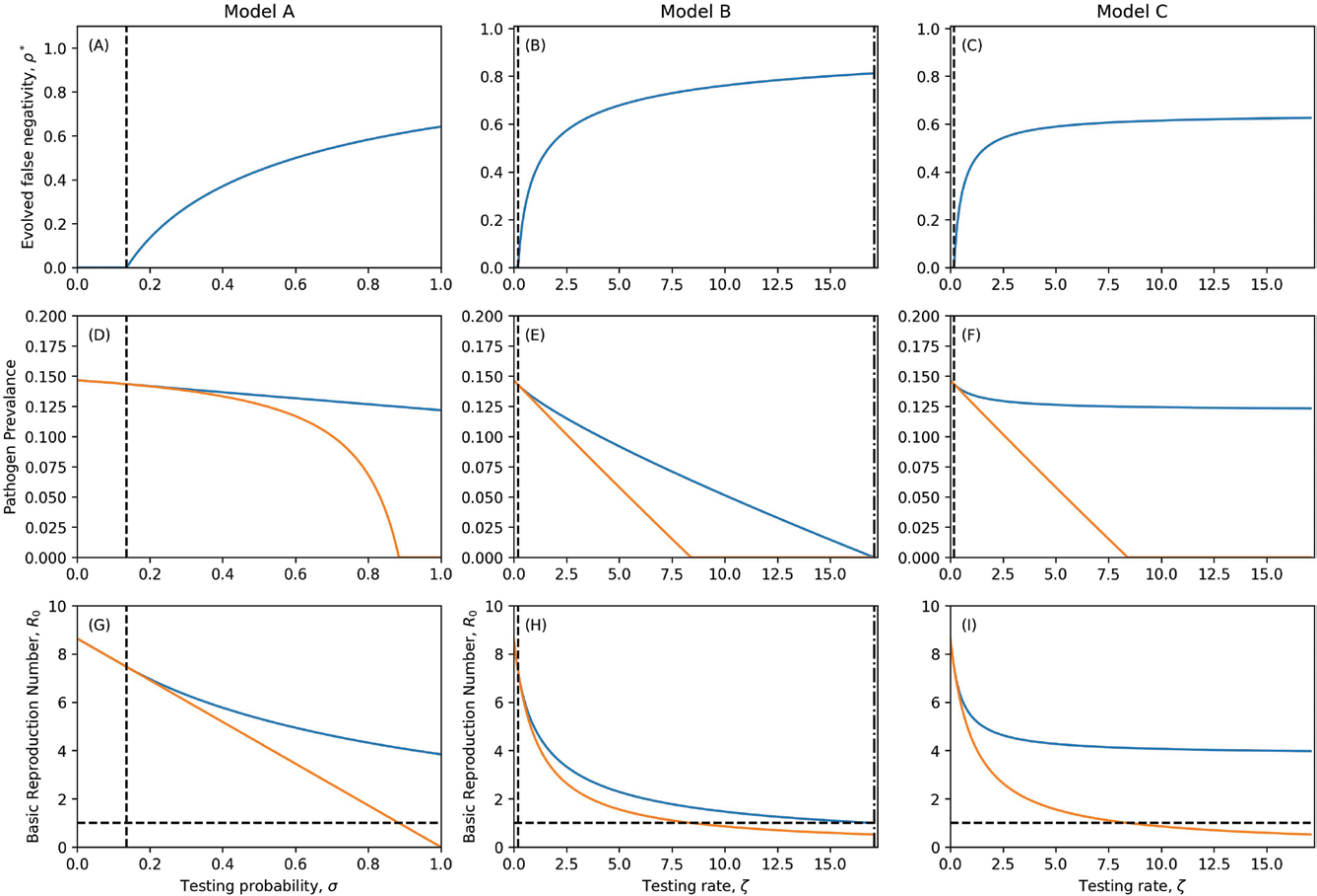
Evolved levels of false negativity under perfect compliance and isolating ($\rho^{*};\;$ A–C), corresponding pathogen prevalence (proportion of hosts infected; D–F) and basic reproduction numbers ($R_{0}$; G–I). The dashed vertical lines denote the level of testing required to select for detection avoidance (ρ>0). Dot-dashed lines indicate pathogen extinction thresholds for the evolved pathogen $\left(R_{0}<1\right)$. In G–I, the horizontal dashed line indicates the extinction threshold $R_{0}=1$. In D–I, the blue curve corresponds to the evolved pathogen and the orange curve to the ancestral state with no detection avoidance (ρ=0). Parameters as in Table 1 except where specified, δ=0, η=1. A colour version of this figure appears in the online version of this article.

Unsurprisingly, more testing (between the minimum and extinction thresholds) selects for greater detection avoidance (Fig. 3A–C). Testing also reduces pathogen prevalence (Fig. 3D–F) and the basic reproduction number $R_{0}$ (Fig. 3G-I), but through the evolution of detection avoidance the pathogen can persist at much higher levels of testing than the pathogen which cannot escape the diagnostic tests. Crucially, from a public health perspective, even if the pathogen evolves detection avoidance, testing still reduces pathogen prevalence. This occurs for two reasons. First, unless detection avoidance is perfect (ρ=1) or isolation measures are completely ineffective (η=0 or δ=0), there is a non-zero probability that a test will be positive and result in reduced transmission due to isolation. Second, detection avoidance is costly, resulting in an intrinsic reduction in transmission.

### Imperfect isolation

**Figure 4.**
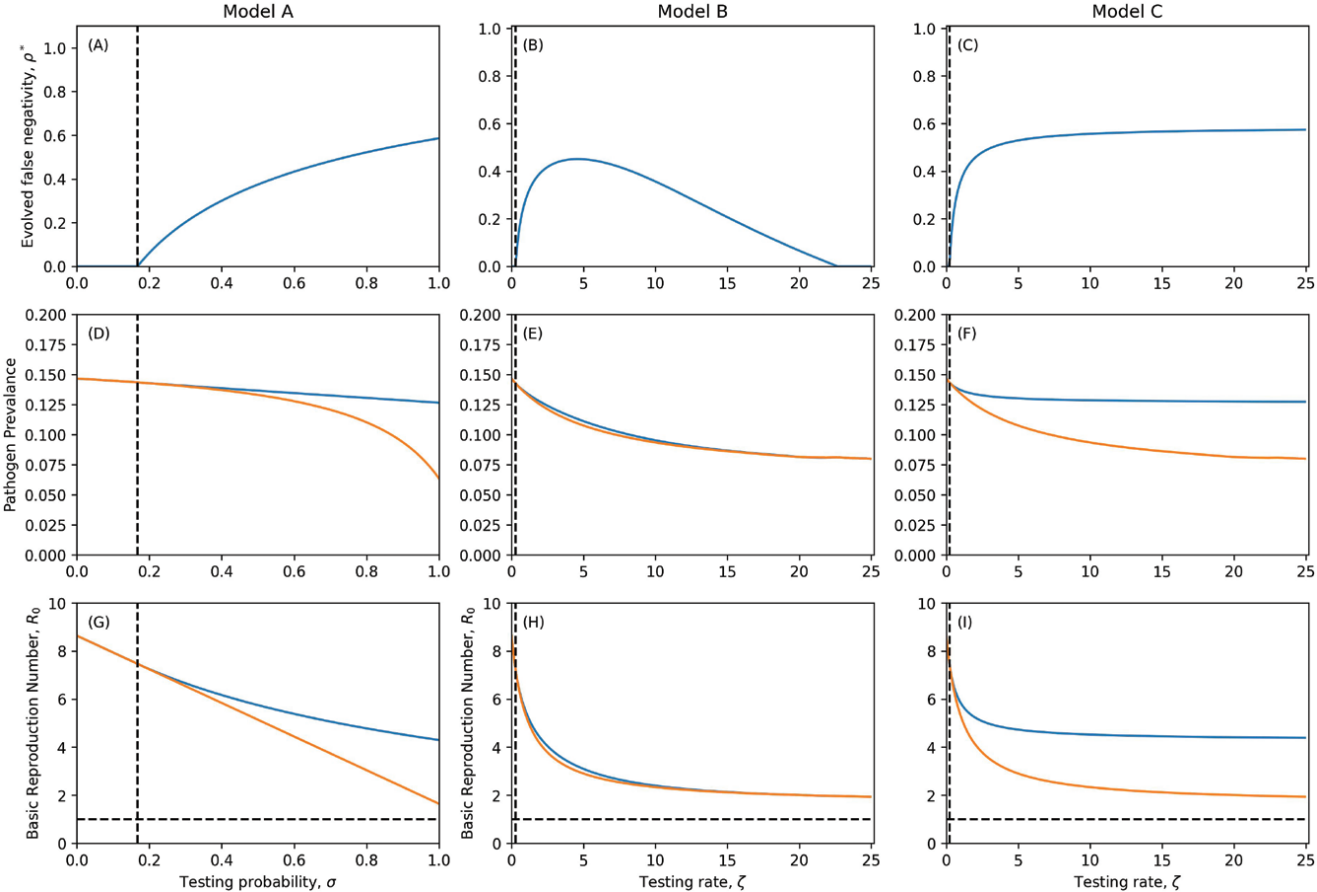
Evolved levels of false negativity with imperfect isolating (A–C), corresponding pathogen prevalence (D–F) and reproduction numbers (G–I). Dashed vertical line denotes level of testing required to select for false-negative tests. Dotted and Dashed line indicates extinction thresholds. In Reproduction number plots a horizontal dashed line indicates the value $R_{0}=1$. In pathogen prevalence and reproduction number plot, blue line indicates prevalence or $R_{0}$ of singular strategy, orange line indicates prevalence or $R_{0}$ in the absence of evolution. Parameters as in Table 1 except where specified, δ=0.1, η=0.9. A colour version of this figure appears in the online version of this article.


In reality, the effectiveness of isolating and compliance with public health measures is imperfect (δ>0, η<1). Naturally, a decrease in compliance or the effectiveness of isolating will shift the thresholds for extinction and the evolution of detection avoidance to higher testing probabilities (Model A) or rates (Models B and C). The testing threshold for the evolution of detection avoidance in Model A is closely related to the perfect compliance/isolating scenario, with


$$\begin{array}{l} \sigma >\frac{-\beta^{\prime }\left(0\right)}{\eta \left(\delta -1\right)\left(\beta \left(0\right)-\beta^{\prime }\left(0\right)\right)} \end{array}$$ (15)

for detection avoidance to evolve. Clearly, when isolation is perfect (η=1 and δ=0), this is equivalent to equation S53. Note that for sufficiently ineffective public health measures (whether in terms of compliance or the strictness of isolation measures), the right-hand side of this inequality is greater than one and so there is no level of testing which selects for detection avoidance. The loss of perfect compliance and isolating has no qualitative effect on the evolution of detection avoidance in Model A (Figs. 3A and 4A) and in Model C, as the testing rate threshold is closely related to the perfect compliance / isolating scenario with

$$\begin{array}{l} \zeta >\frac{\beta^{\prime }\left(0\right)\left(d+\alpha +\gamma \right)}{\beta^{\prime }\left(0\right)\left(-1-\delta \eta +\eta \right)+\eta \beta \left(0\right)\left(\delta -1\right)} \end{array}$$ (16)

for detection avoidance to evolve. As in Model A, when isolation and compliance is perfect, we recover equation S67.

In Model B, detection avoidance evolves provided


$$\begin{array}{lll} & \left(\zeta +d+\alpha +\gamma \right)\left(\left(1+\left(\delta -1\right)\eta \right)\zeta +d+\alpha +\gamma \right)\; & \;\beta^{\prime }\left(0\right)-\eta \zeta \beta \left(0\right)\left(\delta -1\right)\left(d+\alpha +\gamma \right)>0. \end{array}$$ (17)

This inequality is quadratic in the testing rate, ζ, and so can be solved to find upper and lower bounds for when testing selects for detection avoidance. Detection avoidance therefore peaks for an intermediate testing rate in Model B (Fig. 4B), in contrast to the perfect compliance and isolating scenario (Fig. 3B).

This result is somewhat unintuitive, as one might expect that an increase in testing always selects for greater detection avoidance (as in Fig. 3B). However, this does not account for the costs associated with false-negative tests and the ‘leakiness’ that occurs with imperfect isolating. As the testing rate becomes sufficiently large, infected individuals rapidly leave the unaware class. Hence, production of new infections from the aware classes (either due to imperfect isolating or some hosts not isolating) becomes increasingly important, with detection avoidance less advantageous due to the independence between tests.

This can be thought of as a shifting of priorities. When testing is rare, selection favours high transmissibility and low detection avoidance as infected individuals spend most of their time in the unaware class (detection avoidance is not necessary). As testing becomes more common, selection favours detection avoidance as this increases time spent in the unaware class where onwards transmission is greatest, assuming the $R_{0}$ is sufficiently high that testing does not immediately supress the spread of infections. But as testing becomes even more frequent, selection once again favours higher transmission and lower detection avoidance as most infected individuals quickly move into the aware classes even if the pathogen produces false-negative tests, because the result of each test is independent. If an infected individual takes n tests, then the probability that all tests are negative is $\rho^{n}$ which tends to 0 as n→∞ provided ρ≠1, and hence a higher testing rate renders detection avoidance ineffective if the results of each test are independent.

In contrast to perfect isolating and compliance (Fig. 3), imperfect isolating may be insufficient to drive the pathogen extinct, even if testing occurs at a very high rate and there is no detection avoidance (Fig. 4D–F). This is because the pathogen may be completely sustained by infections produced in the aware classes. Our observations indicate that for even slightly imperfect isolating or compliance, the testing rate needed to cause extinction is unreasonably high. We also note that while testing always reduces the prevalence of the pathogen, the imperfect nature of isolating and compliance with public health measures may mean that the prevalence does not drastically reduce (Fig. 4D–F). This indicates that the effectiveness of diagnostic tests for reducing disease prevalence may be limited.

### Stochastic simulations

**Figure 5.**
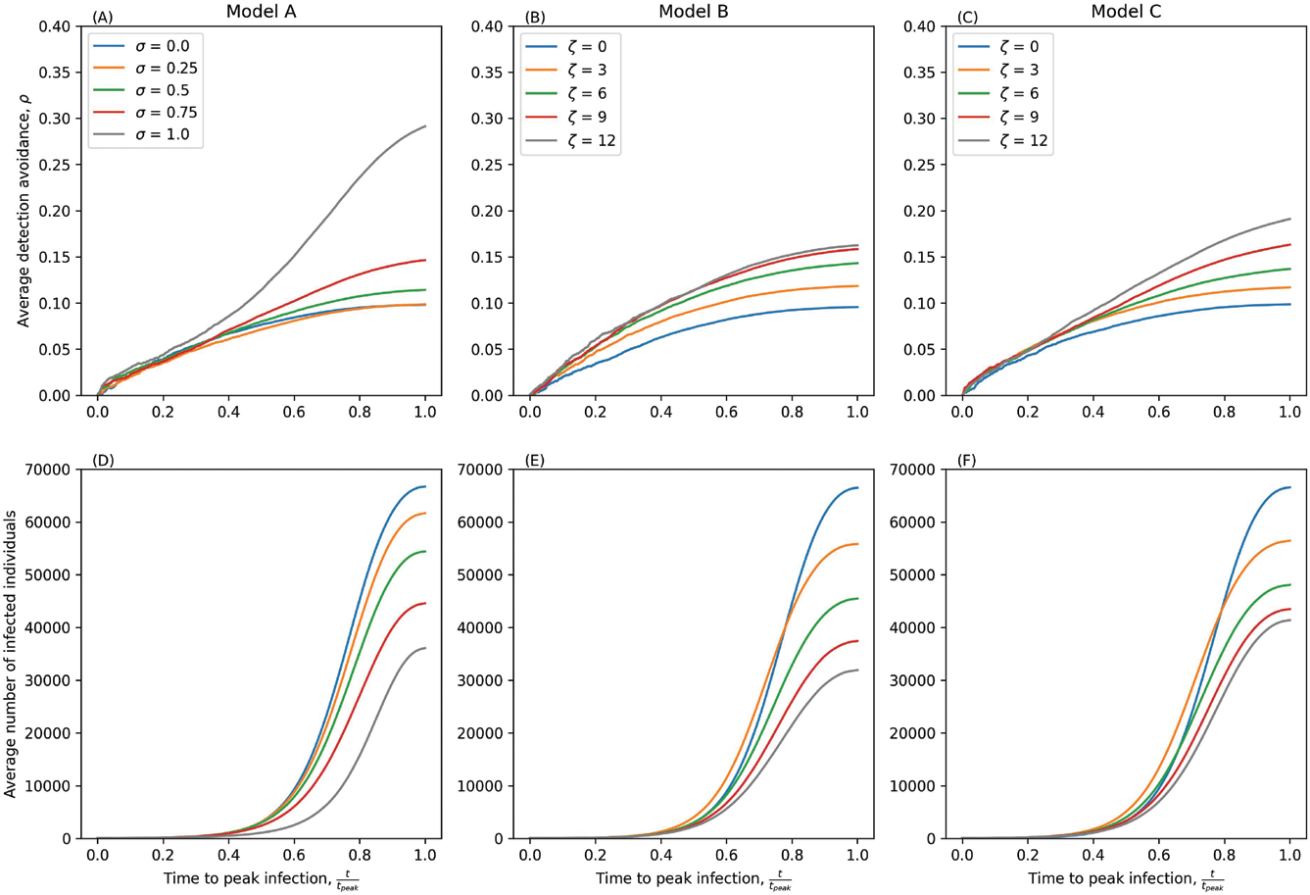
Stochastic simulations of Models A–C with imperfect isolating in a large, non-replenishing population. The first row of figures (A–C) show the average detection avoidance at a given time. The second row of figures (D–F) show the average number of infected individuals. Parameters as in Table 1 except δ=0.1, η=0.9, β=0.0001, N=100,000

Our long-term adaptive dynamics predictions are consistent in stochastic simulations in smaller, finite populations with replenishment, with detection avoidance increasing with greater testing in Models A and C but peaking at intermediate testing in Model B (Supplementary Fig. S1). The short-term evolutionary dynamics in a large but non-replenishing population are broadly similar in Models A and C, with detection avoidance increasing with greater testing (Fig. 5). However, in Model A we do observe a substantial increase in detection avoidance when every individual is tested (σ=1), which does not occur in our adaptive dynamics analysis. This suggests that extreme levels of testing create a very strong short-term selection for detection avoidance, but this recedes over the long term. In Model C we see no significant differences between our adaptive dynamics analysis and the results of stochastic simulations. In Model B, however, selection for detection avoidance saturates as the testing rate increases rather than peaking at intermediate testing rates (Supplementary Fig. S2). This suggests that during the expansion phase of an epidemic, there may still be an advantage to detection avoidance even if hosts are frequently tested with each test having an independent result, as there is still a large pool of susceptible hosts that has yet to be sufficiently depleted. When the time length of the large population simulations is extended, we recover the behaviours seen within the adaptive dynamics framework (Supplementary Fig S3).

## DISCUSSION

The evolution of pathogens in response to human interventions is well documented [1, 4, 5, 8, 10, 20]. Intuitively, efforts to manage infectious diseases naturally select for pathogen variants that can resist or evade interventions. The evolution of antimicrobial resistance (AMR) [1] and vaccine escape [20] are the most prominent examples. Yet pathogens can also evolve in response to NPIs [6]. In particular, there is a growing body of evidence that many pathogens can readily evolve in response to diagnostic testing, but little is known about precisely how diagnostic testing affects selection for detection avoidance. As far as we are aware, only four theoretical studies [4, 9, 14, 16] have considered the evolution of detection avoidance arising from diagnostic testing, and these have focussed on specific pathogens rather than the development of general eco-evolutionary theory of detection avoidance.

Here we have explored how three approaches to diagnostic testing drive the evolution of detection avoidance. When infected individuals may only take a single test (Models A and C), there is a minimum threshold for selection for detection avoidance, above which more testing always results in selection for higher false negativity. In some cases, a sufficiently high level of testing may drive the pathogen extinct. In contrast, when infected individuals regularly test for infection but test results are independent (Model B), more frequent testing does not necessarily lead to selection for higher false negativity. When either isolating or compliance are imperfect, detection avoidance peaks at an intermediate testing rate in Model B, with higher testing rates selecting for low levels of detection avoidance in the long term. In the perfect isolating and compliance scenario, an increase in the testing rate leads to more ‘dead-end’ infections by fully preventing onwards transmission, and hence detection avoidance increases with the testing rate. But in the imperfect isolating and compliance scenario, aware individuals can still produce new infections. Hence, as the testing rate increases, selection shifts from prioritizing transmission before isolating (investment in detection avoidance) to transmission in the aware class (investment in infectivity). However, our stochastic simulations reveal that the latter outcome only occurs in the long term, as detection avoidance instead saturates as the testing rate increases in the short term. This suggests that during the epidemic phase, selection for detection avoidance is higher than during the endemic phase, most likely due to there still being a large pool of susceptible hosts to exploit. This is analogous to virulence evolution, where move virulent strains may have a growth advantage during the early stages of an epidemic, but lose out in the long run when hosts become more scarce (see e.g. [21]).

A possible link between virulence, infectivity and detection has also been proposed as a possible limiting factor for the evolution of virulence, with Kennedy [22] arguing that many human diseases would be expected to experience selection against virulence if disease severity influences human behaviour. The model presented in Kennedy [22] is similar to our model A, under the assumption that all hosts test (i.e. testing probability σ=1) and all aware hosts comply (i.e. compliance probability η=1). Like us, Kennedy finds that detection avoidance often evolves to intermediate levels, assuming there is a trade-off between transmission and likelihood of detection.

Although the evolution of detection avoidance from diagnostic testing presents a significant public health concern, in our models, with the trade-off between diagnostic escape and transmission and no other epidemiological effects (e.g. if detection avoidance correlates with antibody production and therefore affects the potential for reinfection), testing still always reduces overall pathogen prevalence in the population relative to the absence of testing. This happens for two reasons. First, unless the pathogen completely avoids detection, testing inevitably leads to a reduction in transmission as some individuals will isolation. Second, because we assumed that there is a trade-off between false negativity and transmissibility—motivated by evidence from pathogens including *P. falciparum* [14], *C*. *trachomatis* [23] and SARS-CoV-2 [9]—avoiding detection is costly in the absence of testing. Detection avoidance therefore cannot result in an increase in pathogen prevalence relative to the absence of testing. However, the emergence of detection avoidance would clearly result in an increase in pathogen prevalence at a given level of testing.

Our model made a number of key assumptions which should be relaxed and explored in future work. Most importantly, when using an adaptive dynamics framework, which employs a separation of ecological and evolutionary timescales, we were not able to explore selection for detection avoidance during transient epidemic dynamics. However, this approach does provide qualitative insights into the effects of healthcare interventions on the direction of selection (see also [24]), and the adaptive dynamics assumptions were relaxed in our stochastic simulations. We also assumed that the host population was homogeneous, but significant heterogeneity in both disease outcomes and compliance with NPIs is likely in most populations. For example, during the COVID-19 pandemic there was considerable variation in compliance with NPIs [25]. Such heterogeneity may weaken the effects of diagnostic testing on transmission (similar to reducing η or δ in our model), and hence generally weaken selection for detection avoidance. The effects of awareness-driven behaviour changes at the population level can also lead to complex feedback on epidemic dynamics, but these effects have received relatively little attention [26]. Finally, a key assumption in Model B is that the outcome of each test is independent of all others. Hence, repeated testing at a high enough rate would eventually detect most (if not all) infections (provided ρ<1). This was based on the assumption that detection avoidance is probabilistic, but if tests always yield the same result for a given infection, then the peak in detection avoidance at intermediate testing rates does not occur, as observed in Model C. Finally, we did not consider the economic costs or side effects of testing, including the impact of social isolation due to isolating, or the impact of false positive tests on the population, as our focus was on the evolutionary effects of diagnostic testing.

This study was motivated by evidence of detection avoidance arising from diagnostic testing in a variety of pathogens [4, 5, 7, 8, 10, 11]. Rapid antigenic and PCR testing became particularly widespread during the COVID-19 pandemic, and this may signal a shift in attitudes towards (and the availability of) diagnostic tests for other pathogens. Interestingly, as new major variants emerged during the COVID-19 pandemic, a cyclical pattern emerged in spike gene target failures (SGTF) in PCR tests, which allowed for the accurate identification of specific variants [27]. While this did not lead to detection avoidance, clearly the spike gene was under selection (although cycles in SGTF could have arisen due to interference competition; i.e. the Hill-Robertson effect). One might speculate that had the spike gene been the only target of PCR tests, variants with SGTF would have been at an even greater advantage due to detection avoidance. Conversely, the multi-pronged testing regime used in many countries during the COVID-19 pandemic (frequent rapid antigen tests and PCR testing with multiple target genes) is likely a good strategy to help mitigate the emergence of variants that can avoid diagnostic tests.

While theoretical studies of detection avoidance evolution are rare, there are strong analogies with sexually transmitted infections (STIs), such as chlamydia and gonorrhoea, which may have evolved to be inconspicuous to avoid detection by choosy mates [28, 29]. If prospective mates are less likely to choose partners who display signs of infection, then this can create a strong selection for being inconspicuous, similar to the selection for diagnostic testing avoidance. For example, Syphilis, which initially presented as a severely acute and noticeable disease during the middle ages, rapidly evolved to be much more cryptic, likely due to mate-choice effects in humans [30]. Thus, the patterns we see in our model are in good agreement with established evolutionary theory.

Overall, we have shown how the nature of testing, compliance with public health measures to reduce transmission, and the efficacy of isolating measures, can dramatically drive selection for detection avoidance. In particular, our results demonstrate that detection avoidance can readily evolve above a threshold level of testing, but also that selection for detection avoidance can weaken, or even change direction, at higher levels of testing. However, since diagnostic testing always reduces disease prevalence, it is likely to be beneficial despite the evolution of detection avoidance.

## Supplementary Material

eoae018_suppl_Supplementary_Material

## Data Availability

The code used to produce the figures is available on Github here.
