# Supplementary material for: Diagnostic testing and the evolution of detection avoidance by pathogens
Source: Evol Med Public Health. 2024 Aug 27;12(1):248–59. doi: 10.1093/emph/eoae018 (PMC12079369; doi:10.1093/emph/eoae018)
Supplement: eoae018_suppl_Supplementary_Material [file eoae018_suppl_supplementary_material.docx]

Supplementary Material for: Diagnostic testing and the evolution of detection avoidance by pathogens
Jason Wood^1,*^ and Ben Ashby^1,2,3^

1. Department of Mathematical Sciences, University of Bath.
2. Department of Mathematics, Simon Fraser University.
3. The Pacific Institute on Pathogens, Pandemics and Society (PIPPS), Simon Fraser University, Burnaby, BC

[1 Derivation of fitness gradients 2](#_Toc144286569)

[1.1 Model A 2](#_Toc144286570)

[1.2 Model B 3](#_Toc144286571)

[1.3 Model C 3](#_Toc144286572)

[2 Stability analysis of singular strategies 5](#_Toc144286573)

[2.1 Model A 5](#_Toc144286574)

[2.2 Model B 6](#_Toc144286575)

[2.3 Model C 7](#_Toc144286576)

[3 Selection for detection avoidance 8](#_Toc144286577)

[3.1 Model A 8](#_Toc144286578)

[3.1.1 Perfect compliance and quarantining 8](#_Toc144286579)

[3.1.2 Imperfect quarantining 8](#_Toc144286580)

[3.2 Model B 9](#_Toc144286581)

[3.2.1 Perfect compliance and quarantining 9](#_Toc144286582)

[3.2.2 Imperfect quarantining 10](#_Toc144286583)

[3.3 Model C 11](#_Toc144286618)

[3.3.1 Perfect compliance and quarantining 11](#_Toc144286619)

[3.3.2 Imperfect quarantining 11](#_Toc144286620)

[4 Supplementary figures 12](#_Toc144286621)

# Derivation of fitness gradients

## Model A

The rare mutant dynamics for Model A are described by

| $\dot{U_{m}}=\left( \sigma\rho_{m}+\left( 1-\sigma\right) \right)\lambda\left( \rho_{m} \right)S^{*}-\left( d+\alpha+\gamma\right)U_{m}$ | S1 |
| --- | --- |
| $\dot{A_{m}}=\sigma\left( 1-\rho_{m} \right)\left( 1-\eta\right)\lambda\left( \rho_{m} \right)S^{*}-\left( d+\alpha+\gamma\right)A_{m}$ | S2 |
| $\dot{Q_{m}}=\sigma\left( 1-\rho_{m} \right)\eta\lambda\left( \rho_{m} \right)S^{*}-\left( d+\alpha+\gamma\right)Q_{m}$ | S3 |

with Jacobian

| $J=\left( \begin{matrix} \left( \sigma\rho_{m}+\left( 1-\sigma\right) \right)\beta\left( \rho_{m} \right)S^{*}-(d+\alpha+\gamma) & \left( \sigma\rho_{m}+\left( 1-\sigma\right) \right)\beta\left( \rho_{m} \right)S^{*} & \left( \sigma\rho_{m}+\left( 1-\sigma\right) \right)\delta\beta\left( \rho_{m} \right)S^{*} \\ \sigma\left( 1-\rho_{m} \right)\eta\beta\left( \rho_{m} \right)S^{*} & \sigma\left( 1-\rho_{m} \right)\eta\beta\left( \rho_{m} \right)S^{*}-(d+\alpha+\gamma) & \sigma\left( 1-\rho_{m} \right)\eta\delta\beta\left( \rho_{m} \right)S^{*} \\ \sigma\left( 1-\rho_{m} \right)(1-\eta)\beta\left( \rho_{m} \right)S^{*} & \sigma\left( 1-\rho_{m} \right)(1-\eta)\beta\left( \rho_{m} \right)S^{*} & \sigma\left( 1-\rho_{m} \right)(1-\eta)\delta\beta\left( \rho_{m} \right)S^{*}-(d+\alpha+\gamma) \end{matrix} \right)$ | S4 |
| --- | --- |

From this we can derive the next generation matrix, $NG=FV^{-1}$, where:

| $F=\left( \begin{matrix} \left( \sigma\rho_{m}+\left( 1-\sigma\right) \right)\beta\left( \rho_{m} \right)S^{*} & \left( \sigma\rho_{m}+\left( 1-\sigma\right) \right)\beta\left( \rho_{m} \right)S^{*} & \left( \sigma\rho_{m}+\left( 1-\sigma\right) \right)\delta\beta\left( \rho_{m} \right)S^{*} \\ \sigma\left( 1-\rho_{m} \right)\eta\beta\left( \rho_{m} \right)S^{*} & \sigma\left( 1-\rho_{m} \right)\eta\beta\left( \rho_{m} \right)S^{*} & \sigma\left( 1-\rho_{m} \right)\eta\delta\beta\left( \rho_{m} \right)S^{*} \\ \sigma\left( 1-\rho_{m} \right)(1-\eta)\beta\left( \rho_{m} \right)S^{*} & \sigma\left( 1-\rho_{m} \right)(1-\eta)\beta\left( \rho_{m} \right)S^{*} & \sigma\left( 1-\rho_{m} \right)(1-\eta)\delta\beta\left( \rho_{m} \right)S^{*} \end{matrix} \right)$ | S5 |
| --- | --- |
| $V=F-J=\left( \begin{matrix} d+\alpha+\gamma& 0 & 0 \\ 0 & d+\alpha+\gamma& 0 \\ 0 & 0 & d+\alpha+\gamma\end{matrix} \right)$ | S6 |
| $V^{-1}=\left( \begin{matrix} \frac{1}{d+\alpha+\gamma} & 0 & 0 \\ 0 & \frac{1}{d+\alpha+\gamma} & 0 \\ 0 & 0 & \frac{1}{d+\alpha+\gamma} \end{matrix} \right)$ | S7 |
| $NG=\left( \begin{matrix} \frac{\left( \sigma\rho_{m}+\left( 1-\sigma\right) \right)\beta\left( \rho_{m} \right)S^{*}}{d+\alpha+\gamma} & \frac{\left( \sigma\rho_{m}+\left( 1-\sigma\right) \right)\beta\left( \rho_{m} \right)S^{*}}{d+\alpha+\gamma} & \frac{\left( \sigma\rho_{m}+\left( 1-\sigma\right) \right)\delta\beta\left( \rho_{m} \right)S^{*}}{d+\alpha+\gamma} \\ \frac{\sigma\left( 1-\rho_{m} \right)\eta\beta\left( \rho_{m} \right)S^{*}}{d+\alpha+\gamma} & \frac{\sigma\left( 1-\rho_{m} \right)\eta\beta\left( \rho_{m} \right)S^{*}}{d+\alpha+\gamma} & \frac{\sigma\left( 1-\rho_{m} \right)\eta\delta\beta\left( \rho_{m} \right)S^{*}}{d+\alpha+\gamma} \\ \frac{\sigma\left( 1-\rho_{m} \right)(1-\eta)\beta\left( \rho_{m} \right)S^{*}}{d+\alpha+\gamma} & \frac{\sigma\left( 1-\rho_{m} \right)(1-\eta)\beta\left( \rho_{m} \right)S^{*}}{d+\alpha+\gamma} & \frac{\sigma\left( 1-\rho_{m} \right)(1-\eta)\delta\beta\left( \rho_{m} \right)S^{*}}{d+\alpha+\gamma} \end{matrix} \right)$ | S8 |

The largest eigenvalue of the next generation matrix, and thus our sign equivalent expression for fitness is then,

| $r\left( \rho_{m},\rho\right)=-\frac{\beta\left( \rho_{m} \right)S^{*}\left( \rho_{m}\eta\sigma\delta-\rho_{m}\eta\sigma-\eta\sigma\delta+\eta\sigma-1 \right)}{d+\alpha+\gamma}-1$ | S9 |
| --- | --- |

And hence the fitness gradient is,

| $\mathcal{F}\left( \rho\right)=\left. \frac{dr}{d\rho_{m}} \right\vert_{\rho_{m}=\rho} =\frac{-S^{*}\left( \left( -1+\eta\left( \delta-1 \right)\left( \rho-1 \right)\sigma\right)\frac{d\beta(\rho)}{d\rho}+\beta\left( \rho\right)\eta\sigma\left( \delta-1 \right) \right)}{d+\alpha+\gamma}$ | S10 |
| --- | --- |

## Model B

The rare mutant dynamics are described by

| $\dot{U_{m}}=\lambda\left( \rho_{m} \right)S^{*}-\zeta\left( 1-\rho_{m} \right)U_{m}-\left( d+\alpha+\gamma\right)U_{m}$ | S11 |
| --- | --- |
| $\dot{A_{m}}=\zeta\left( 1-\rho_{m} \right)\left( 1-\eta\right)U_{m}-\left( d+\alpha+\gamma\right)A_{m}$ | S12 |
| $\dot{Q_{m}}=\zeta\left( 1-\rho_{m} \right)\eta U_{m}-\left( d+\alpha+\gamma\right)Q_{m}$ | S13 |

with Jacobian

| $J=\left( \begin{matrix} \beta\left( \rho_{m} \right)S^{*}-\zeta\left( 1-\rho_{m} \right)-\left( d+\alpha+\gamma\right) & \beta\left( \rho_{m} \right)S^{*} & \delta\beta\left( \rho_{m} \right)S^{*} \\ \zeta\left( 1-\rho_{m} \right)\eta& -(d+\alpha+\gamma) & 0 \\ \zeta\left( 1-\rho_{m} \right)\left( 1-\eta\right) & 0 & -(d+\alpha+\gamma) \end{matrix} \right)$ | S14 |
| --- | --- |

Using the same method as above, we can calculate the next generation matrix, $NG=FV^{-1}$, where

| $F=\left( \begin{matrix} \beta\left( \rho_{m} \right)S^{*} & \beta\left( \rho_{m} \right)S^{*} & \delta\beta\left( \rho_{m} \right)S^{*} \\ 0 & 0 & 0 \\ 0 & 0 & 0 \end{matrix} \right)$ | S15 |
| --- | --- |
| $V=F-J=\left( \begin{matrix} \zeta\left( 1-\rho_{m} \right)+\left( d+\alpha+\gamma\right) & 0 & 0 \\ -\zeta\left( 1-\rho_{m} \right)\eta& (d+\alpha+\gamma) & 0 \\ -\zeta\left( 1-\rho_{m} \right)\left( 1-\eta\right) & 0 & (d+\alpha+\gamma) \end{matrix} \right)$ | S16 |
| $V^{-1}=\left( \begin{matrix} \frac{-1}{\zeta\left( 1-\rho_{m} \right)+\left( d+\alpha+\gamma\right)} & 0 & 0 \\ \frac{\zeta\left( 1-\rho_{m} \right)\eta}{\left( d+\alpha+\gamma\right)\left( \zeta\left( 1-\rho_{m} \right)+\left( d+\alpha+\gamma\right) \right)} & \frac{1}{d+\alpha+\gamma} & 0 \\ \frac{\zeta\left( 1-\rho_{m} \right)(\eta-1)}{\left( d+\alpha+\gamma\right)\left( \zeta\left( 1-\rho_{m} \right)+\left( d+\alpha+\gamma\right) \right)} & 0 & \frac{1}{d+\alpha+\gamma} \end{matrix} \right)$ | S17 |
| $NG=\left( \begin{matrix} \frac{\delta\beta\left( \rho_{m} \right)S^{*}\zeta\left( \rho_{m}-1 \right)\eta}{\left( d+\alpha+\gamma\right)\left( \zeta\left( \rho_{m}-1 \right)-\left( d+\alpha+\gamma\right) \right)}-\frac{\beta\left( \rho_{m} \right)S^{*}\zeta\left( \rho_{m}-1 \right)\left( \eta-1 \right)}{\left( d+\alpha+\gamma\right)\left( \zeta\left( \rho_{m}-1 \right)-\left( d+\alpha+\gamma\right) \right)}-\frac{\beta\left( \rho_{m} \right)S^{*}}{\zeta\left( \rho_{m}-1 \right)-\left( d+\alpha+\gamma\right)} & \frac{\beta\left( \rho_{m} \right)S^{*}}{d+\alpha+\gamma} & \frac{\delta\beta\left( \rho_{m} \right)S^{*}}{d+\alpha+\gamma} \\ 0 & 0 & 0 \\ 0 & 0 & 0 \end{matrix} \right)$ | S18 |

From which we can derive an expression which is sign equivalent to fitness minus one

| $r\left( \rho_{m},\rho\right)=\frac{\beta\left( \rho_{m} \right)S^{*}(\delta\zeta\eta\rho_{m}-\rho_{m}\zeta\eta-\delta\zeta\eta+\rho_{m}\zeta+\zeta\eta-\left( d+\alpha+\gamma\right)-\zeta)}{\left( d+\alpha+\gamma\right)\left( \zeta\left( \rho_{m}-1 \right)-\left( d+\alpha+\gamma\right) \right)}-1$ | S19 |
| --- | --- |

and hence the fitness gradient is:

| $\mathcal{F}(\rho)=\frac{1}{\left( d+\alpha+\gamma\right)\left( \zeta\left( 1-\rho\right)+d+\alpha+\gamma\right)^{2}}\left( \left( \left( \zeta\left( 1-\rho\right)+d+\alpha+\gamma\right)\left( -\left( 1+\left( \delta-1 \right)\eta\right)\left( \rho-1 \right)\zeta+d+\alpha+\gamma\right)\beta'\left( \rho\right)-\eta\zeta\beta(\rho)\left( \delta-1 \right)\left( d+\alpha+\gamma\right) \right)S^{*} \right)$ | S20 |
| --- | --- |

## Model C

The mutant dynamics are described by

| $\dot{U_{m}}=\rho_{m}\lambda\left( \rho_{m} \right)S^{*}-\left( d+\alpha+\gamma\right)U_{m}$ | S21 |
| --- | --- |
| $\dot{I_{m}}=\left( 1-\rho_{m} \right)\lambda\left( \rho_{m} \right)S^{*}-\zeta I_{m}-\left( d+\alpha+\gamma\right)I_{m}$ | S22 |
| $\dot{A_{m}}=\zeta\left( 1-\eta\right)I_{m}-\left( d+\alpha+\gamma\right)A_{m}$ | S23 |
| $\dot{Q_{m}}=\zeta\eta I_{m}-\left( d+\alpha+\gamma\right)Q_{m}$ | S24 |

with Jacobian

| $J=\left( \begin{matrix} \rho_{m}\beta\left( \rho_{m} \right)S^{*}-\left( d+\alpha+\gamma\right) & \rho_{m}\beta\left( \rho_{m} \right)S^{*} & \rho\beta\left( \rho_{m} \right)S^{*} & \delta\rho_{m}\beta\left( \rho_{m} \right)S^{*} \\ {(1-\rho}_{m})\beta\left( \rho_{m} \right)S^{*} & {(1-\rho}_{m})\beta\left( \rho_{m} \right)S^{*}-\zeta-(d+\alpha+\gamma) & {(1-\rho}_{m})\beta\left( \rho_{m} \right)S^{*} & {(1-\rho}_{m})\delta\beta\left( \rho_{m} \right)S^{*} \\ 0 & \zeta(1-\eta) & -(d+\alpha+\gamma) & 0 \\ 0 & \zeta\eta& 0 & -(d+\alpha+\gamma) \end{matrix} \right)$ | S25 |
| --- | --- |

From this we can derive the next generation matrix, $NG=FV^{-1}$, where:

| $F=\left( \begin{matrix} \rho_{m}\beta\left( \rho_{m} \right)S^{*} & \rho_{m}\beta\left( \rho_{m} \right)S^{*} & \rho\beta\left( \rho_{m} \right)S^{*} & \delta\rho_{m}\beta\left( \rho_{m} \right)S^{*} \\ {(1-\rho}_{m})\beta\left( \rho_{m} \right)S^{*} & {(1-\rho}_{m})\beta\left( \rho_{m} \right)S^{*} & {(1-\rho}_{m})\beta\left( \rho_{m} \right)S^{*} & {(1-\rho}_{m})\delta\beta\left( \rho_{m} \right)S^{*} \\ 0 & 0 & 0 & 0 \\ 0 & 0 & 0 & 0 \end{matrix} \right)$ | S26 |
| --- | --- |
| $V=F-J=\left( \begin{matrix} d+\alpha+\gamma& 0 & 0 & 0 \\ 0 & \zeta+d+\alpha+\gamma& 0 & 0 \\ 0 & -\zeta(1-\eta) & d+\alpha+\gamma& 0 \\ 0 & -\zeta\eta& 0 & d+\alpha+\gamma\end{matrix} \right)$ | S27 |
| $V^{-1}=\left( \begin{matrix} \frac{1}{d+\alpha+\gamma} & 0 & 0 & 0 \\ 0 & \frac{1}{\zeta+d+\alpha+\gamma} & 0 & 0 \\ 0 & \frac{-\zeta(1-\eta)}{(\zeta+d+\alpha+\gamma)(d+\alpha+\gamma)} & \frac{1}{d+\alpha+\gamma} & 0 \\ 0 & \frac{\zeta\eta}{(\zeta+d+\alpha+\gamma)(d+\alpha+\gamma)} & 0 & \frac{1}{d+\alpha+\gamma} \end{matrix} \right)$ | S28 |
| $NG=\left( \begin{matrix} \frac{\rho_{m}\beta\left( \rho_{m} \right)S^{*}}{d+\alpha+\gamma} & \frac{\rho_{m}\beta\left( \rho_{m} \right)S^{*}(\left( 1+\left( \delta-1 \right)\eta\right)\zeta+d+\alpha+\gamma)}{(d+\alpha+\gamma)(\zeta+d+\alpha+\gamma)} & \frac{\rho_{m}\beta\left( \rho_{m} \right)S^{*}}{d+\alpha+\gamma} & \frac{\delta\rho_{m}\beta\left( \rho_{m} \right)S^{*}}{d+\alpha+\gamma} \\ \frac{(1-\rho_{m})\beta\left( \rho_{m} \right)S^{*}}{d+\alpha+\gamma} & \frac{{(1-\rho}_{m})\beta\left( \rho_{m} \right)S^{*}(\left( 1+\left( \delta-1 \right)\eta\right)\zeta+d+\alpha+\gamma)}{(d+\alpha+\gamma)(\zeta+d+\alpha+\gamma)} & \frac{{(1-\rho}_{m})\beta\left( \rho_{m} \right)S^{*}}{d+\alpha+\gamma} & \frac{\delta(1-\rho_{m})\beta\left( \rho_{m} \right)S^{*}}{d+\alpha+\gamma} \\ 0 & 0 & 0 & 0 \\ 0 & 0 & 0 & 0 \end{matrix} \right)$ | S29 |

The largest eigenvalue of the next generation matrix, and thus our sign equivalent expression for fitness is then,

| $r\left( \rho_{m},\rho\right)=-\frac{S^{*}\left( \left( -1+\left( -1+\rho_{m} \right)\left( \delta-1 \right)\eta\right)\zeta-\left( d+\alpha+\gamma\right) \right)\beta\left( \rho_{m} \right)-(d+\alpha+\gamma)(\zeta+d+\alpha+\gamma)}{(d+\alpha+\gamma)(\zeta+d+\alpha+\gamma)}$ | S30 |
| --- | --- |

And hence the fitness gradient is,

| $\mathcal{F}(\rho)=\frac{\left( \left( -1+\left( -1+\rho\right)\left( \delta-1 \right)\eta\right)\zeta-\left( d+\alpha+\gamma\right)) \beta^{'}\left( \rho\right)+\eta\zeta\beta\left( \rho\right)\left( \delta-1 \right) \right)S^{*}}{(d+\alpha+\gamma)(\zeta+d+\alpha+\gamma)}$ | S31 |
| --- | --- |

# Stability analysis of singular strategies

## Model A

The fitness gradient for model A is,

| $\mathcal{F}(\rho)=\frac{-S^{*}\left( \left( -1+\eta\left( \delta-1 \right)\left( \rho-1 \right)\sigma\right)\frac{d\beta(\rho)}{d\rho}+\beta\left( \rho\right)\eta\sigma\left( \delta-1 \right) \right)}{d+\alpha+\gamma}$ | S32 |
| --- | --- |

and the density of susceptibles at steady state is,

| $S^{*}=\frac{-\left( d+\alpha+\gamma\right)}{\left( -1+\eta\left( \delta-1 \right)\left( -1+\rho\right)\sigma\right)\beta\left( \rho\right)}$ | S33 |
| --- | --- |

with derivative

| $\frac{dS}{d\rho}=\frac{-\left( d+\alpha+\gamma\right)\eta\left( \delta-1 \right)\sigma}{\left( -1+\eta\left( \delta-1 \right)\left( -1+\rho\right)\sigma\right)^{2}\beta\left( \rho\right)}-\frac{-\left( d+\alpha+\gamma\right)\frac{d\beta(\rho)}{d\rho}}{\left( -1+\eta\left( \delta-1 \right)\left( -1+\rho\right)\sigma\right)\beta\left( \rho\right)^{2}}$ | S34 |
| --- | --- |

At the singular strategy the fitness gradient is zero, and hence we know,

| $\left. \frac{d\beta\left( \rho\right)}{d\rho} \right\vert_{\rho=\rho^{*}}=\frac{-\sigma\eta\left( \delta-1 \right)\beta(\rho^{*})}{-1+\eta\left( -1+\rho^{*} \right)\left( \delta-1 \right)\sigma}$ | S35 |
| --- | --- |

Hence, substituting in expression S35 into S34 gives,

| $\left. \frac{dS}{d\rho} \right\vert_{\rho=\rho^{*}}=\frac{\left( d+\alpha+\gamma\right)\eta\left( \delta-1 \right)\sigma}{\left( -1+\eta\left( \delta-1 \right)\left( -1+\rho^{*} \right)\sigma\right)^{2}\beta\left( \rho^{*} \right)}-\frac{\left( d+\alpha+\gamma\right)\sigma\eta\left( \delta-1 \right)}{\left( -1+\eta\left( \delta-1 \right)\left( -1+\rho^{*} \right)\sigma\right)^{2}\beta\left( \rho^{*} \right)}=0$ | S36 |
| --- | --- |

The convergence stability of a singular strategy $\rho^{*}$ is determined by the mutual invaisability $M$, and the evolutionary stability $E$, where $M=\left. \frac{\partial^{2}r(\rho_{m},\rho)}{\partial\rho\partial\rho_{m}} \right|_{\rho=\rho_{m}=\rho^{*}}$ and $E=\left. \frac{\partial^{2}r(\rho_{m},\rho)}{\partial\rho_{m}^{2}} \right|_{\rho=\rho_{m}=\rho^{*}}$. A singular strategy is convergence stable when $E+M<0$ and evolutionarily stable when $E<0$. Here,

| $M=\left. \frac{\partial^{2}r\left( \rho_{m},\rho\right)}{\partial\rho\partial\rho_{m}} \right\vert_{\rho=\rho_{m}=\rho^{*}}=\frac{-\left( \left( -1+\eta\left( \delta-1 \right)\left( \rho^{*}-1 \right)\sigma\right)\frac{d\beta(\rho)}{d\rho}\left. \right\vert_{\rho^{*}}+\beta\left( \rho^{*} \right)\eta\sigma\left( \delta-1 \right) \right)}{d+\alpha+\gamma}\left. \frac{dS}{d\rho} \right\vert_{\rho=\rho^{*}}=0$ | S37 |
| --- | --- |

Hence, any singular strategy is convergence stable if it is evolutionarily stable.

## Model B

The fitness gradient in Model B is,

| $\mathcal{F}(\rho)=\frac{1}{\left( d+\alpha+\gamma\right)\left( \zeta\left( 1-\rho\right)+d+\alpha+\gamma\right)^{2}}\left( \left( \left( \zeta\left( 1-\rho\right)+d+\alpha+\gamma\right)\left( -\left( 1+\left( \delta-1 \right)\eta\right)\left( \rho-1 \right)\zeta+d+\alpha+\gamma\right)\beta'\left( \rho\right)-\eta\zeta\beta(\rho)\left( \delta-1 \right)\left( d+\alpha+\gamma\right) \right)S^{*} \right)$ | S38 |
| --- | --- |

And the density of susceptibles is,

| $S=\frac{(\zeta\left( 1-\rho\right)+d+\alpha+\gamma)(d+\alpha+\gamma)}{\beta\left( \rho\right)\left( -1\left( 1+\left( \delta-1 \right)\eta\right)\left( -1+\rho\right)\zeta+d+\alpha+\gamma\right)}$ | S39 |
| --- | --- |

with derivative

| $\frac{dS}{d\rho}=\frac{-1}{\beta\left( \rho\right)^{2}\left( -1\left( -1+\rho\right)\left( 1+\left( \delta-1 \right)\eta\right)\zeta+d+\alpha+\gamma\right)^{2}}(\left( \zeta\left( 1-\rho\right)+d+\alpha+\gamma\right)\left( -1\left( -1+\rho\right)\left( 1+\left( \delta-1 \right)\eta\right)\zeta+d+\alpha+\gamma\right)\frac{d\beta\left( \rho\right)}{d\rho}-\eta\zeta\beta\left( \rho\right)\left( d+\alpha+\gamma\right)\left( \delta-1 \right))(d+\alpha+\gamma))$ | S40 |
| --- | --- |

At the singular strategy $\rho^{*}$, the fitness gradient is 0, and hence,

| $\left. \frac{d\beta\left( \rho\right)}{d\rho} \right\vert_{\rho=\rho^{*}}=\frac{\eta\zeta\beta(\rho^{*})(\delta-1)(d+\alpha+\gamma)}{\left( \zeta\left( 1-\rho^{*} \right)+d+\alpha+\gamma\right)\left( -1\left( 1+\left( \delta-1 \right)\eta\right)\left( -1+\rho^{*} \right)\zeta+d+\alpha+\gamma\right)}$ | S41 |
| --- | --- |

Substituting S41 into S40, we obtain

| $\left. \frac{dS}{d\rho} \right\vert_{\rho=\rho^{*}}=\frac{-1}{\beta\left( \rho^{*} \right)^{2}\left( -1\left( -1+\rho^{*} \right)\left( 1+\left( \delta-1 \right)\eta\right)\zeta+d+\alpha+\gamma\right)^{2}}(\left( \zeta\left( 1-\rho^{*} \right)+d+\alpha+\gamma\right)\left( -1\left( -1+\rho^{*} \right)\left( 1+\left( \delta-1 \right)\eta\right)\zeta+d+\alpha+\gamma\right)\frac{\eta\zeta\beta(\rho^{*})(\delta-1)(d+\alpha+\gamma)}{\left( \zeta\left( 1-\rho^{*} \right)+d+\alpha+\gamma\right)\left( -1\left( 1+\left( \delta-1 \right)\eta\right)\left( -1+\rho^{*} \right)\zeta+d+\alpha+\gamma\right)}-\eta\zeta\beta\left( \rho^{*} \right)\left( d+\alpha+\gamma\right)\left( \delta-1 \right))(d+\alpha+\gamma))$ | S42 |
| --- | --- |
| $\left. \frac{dS}{d\rho} \right\vert_{\rho=\rho^{*}}=\frac{-1}{\beta\left( \rho^{*} \right)^{2}\left( -1\left( -1+\rho^{*} \right)\left( 1+\left( \delta-1 \right)\eta\right)\zeta+d+\alpha+\gamma\right)^{2}}(\eta\zeta\beta(\rho^{*})(\delta-1)(d+\alpha+\gamma)-\eta\zeta\beta\left( \rho^{*} \right)\left( d+\alpha+\gamma\right)\left( \delta-1 \right))(d+\alpha+\gamma))=0$ | S43 |

Here,

| $M=\left. \frac{\partial^{2}r\left( \rho_{m},\rho\right)}{\partial\rho\partial\rho_{m}} \right\vert_{\rho=\rho_{m}=\rho^{*}}=\frac{1}{\left( d+\alpha+\gamma\right)\left( \zeta\left( 1-\rho\right)+d+\alpha+\gamma\right)^{2}}\left( \left( \left( \zeta\left( 1-\rho\right)+d+\alpha+\gamma\right)\left( -\left( 1+\left( \delta-1 \right)\eta\right)\left( \rho-1 \right)\zeta+d+\alpha+\gamma\right)\beta'\left( \rho\right)-\eta\zeta\beta(\rho)\left( \delta-1 \right)\left( d+\alpha+\gamma\right) \right) \right)\left. \frac{dS}{d\rho} \right\vert_{\rho=\rho^{*}}=0$ | S44 |
| --- | --- |

Hence any evolutionarily stable singular strategy is also convergent stable.

## Model C

The fitness gradient in Model C is,

| $\mathcal{F}(\rho)=\frac{\left( \left( -1+\left( -1+\rho\right)\left( \delta-1 \right)\eta\right)\zeta-\left( d+\alpha+\gamma\right)) \beta^{'}\left( \rho\right)+\eta\zeta\beta\left( \rho\right)\left( \delta-1 \right) \right)S^{*}}{(d+\alpha+\gamma)(\zeta+d+\alpha+\gamma)}$ | S45 |
| --- | --- |

And the density of susceptibles is,

| $S=\frac{(\zeta+d+\alpha+\gamma)(d+\alpha+\gamma)}{\left( \left( 1-\left( \delta-1 \right)\left( -1+\rho\right)\eta\right)\zeta+d+\alpha+\gamma\right)\beta\left( \rho\right)}$ | S46 |
| --- | --- |

With derivative

| $\frac{dS}{d\rho}=\frac{\left( \left( \left( 1-\left( \delta-1 \right)\left( -1+\rho\right)\eta\right)\zeta+d+\alpha+\gamma\right)\beta^{'}\left( \rho\right)-\eta\zeta\beta\left( \rho\right)\left( \delta-1 \right) \right)\left( \zeta+d+\alpha+\gamma\right)\left( d+\alpha+\gamma\right)}{\left( \left( 1-\left( \delta-1 \right)\left( -1+\rho\right)\eta\right)\zeta+d+\alpha+\gamma\right)^{2}\beta\left( \rho\right)^{2}}$ | S45 |
| --- | --- |

At the singular strategy $\rho^{*}$, the fitness gradient is 0, and hence,

| $\left. \frac{d\beta\left( \rho\right)}{d\rho} \right\vert_{\rho=\rho^{*}}=\frac{\eta\zeta\beta(\rho^{*})(\delta-1)}{\left( 1-\left( -1+\rho^{*} \right)\left( \delta-1 \right)\eta\right)\zeta+d+\alpha+\gamma}$ | S46 |
| --- | --- |

Substituting S46 into S45, we obtain

| $\left. \frac{dS}{d\rho} \right\vert_{\rho=\rho^{*}}=\frac{\left( \left( \left( 1-\left( \delta-1 \right)\left( -1+\rho^{*} \right)\eta\right)\zeta+d+\alpha+\gamma\right)\frac{\eta\zeta\beta(\rho^{*})(\delta-1)}{\left( 1-\left( -1+\rho^{*} \right)\left( \delta-1 \right)\eta\right)\zeta+d+\alpha+\gamma}-\eta\zeta\beta\left( \rho^{*} \right)\left( \delta-1 \right) \right)\left( \zeta+d+\alpha+\gamma\right)\left( d+\alpha+\gamma\right)}{\left( \left( 1-\left( \delta-1 \right)\left( -1+\rho^{*} \right)\eta\right)\zeta+d+\alpha+\gamma\right)^{2}\beta\left( \rho^{*} \right)^{2}}$ | S47 |
| --- | --- |
| $\left. \frac{dS}{d\rho} \right\vert_{\rho=\rho^{*}}=\frac{\left( \eta\zeta\beta(\rho^{*})(\delta-1)-\eta\zeta\beta\left( \rho^{*} \right)\left( \delta-1 \right) \right)\left( \zeta+d+\alpha+\gamma\right)\left( d+\alpha+\gamma\right)}{\left( \left( 1-\left( \delta-1 \right)\left( -1+\rho^{*} \right)\eta\right)\zeta+d+\alpha+\gamma\right)^{2}\beta\left( \rho^{*} \right)^{2}}=0$ | S48 |

Here

| $M=\left. \frac{\partial^{2}r\left( \rho_{m},\rho\right)}{\partial\rho\partial\rho_{m}} \right\vert_{\rho=\rho^{*}}=\frac{\left( \left( -1+\left( -1+\rho\right)\left( \delta-1 \right)\eta\right)\zeta-\left( d+\alpha+\gamma\right)) \beta^{'}\left( \rho\right)+\eta\zeta\beta\left( \rho\right)\left( \delta-1 \right) \right)}{(d+\alpha+\gamma)(\zeta+d+\alpha+\gamma)}\left. \frac{dS}{d\rho} \right\vert_{\rho=\rho^{*}}=0$ | S49 |
| --- | --- |

Hence any evolutionarily stable singular strategy is also convergence stable.

# Selection for detection avoidance

## Model A

### Perfect compliance and quarantining

The fitness gradient for model A when $\delta=0, \eta=1$ is,

| $\mathcal{F}(\rho)=\frac{-S^{*}\left( \left( -1-\left( \rho-1 \right)\sigma\right)\frac{d\beta(\rho)}{d\rho}-\beta\left( \rho\right)\sigma\right)}{d+\alpha+\gamma}$ | S50 |
| --- | --- |

There is selection for detection avoidance when $\mathcal{F}\left( 0 \right)>0$:

| $\mathcal{F}\left( 0 \right)=\frac{-S^{*}\left( \left( -1-\left( 0-1 \right)\sigma\right)\left. \frac{d\beta\left( \rho\right)}{d\rho} \right\vert_{\rho=0}-\beta\left( 0 \right)\sigma\right)}{d+\alpha+\gamma}=\frac{-S^{*}}{d+\alpha+\gamma}\left( \left( \sigma-1 \right)\left. \frac{d\beta\left( \rho\right)}{d\rho} \right\vert_{\rho=0}-\beta\left( 0 \right)\sigma\right)>0$ | S51 |
| --- | --- |

Hence,

| $\left( \sigma-1 \right)\left. \frac{d\beta\left( \rho\right)}{d\rho} \right\vert_{\rho=0}-\beta\left( 0 \right)\sigma<0$ | S52 |
| --- | --- |

We can rearrange S52 in terms of $\sigma$ to find the minimum testing probability needed for detection avoidance to evolve, which is:

| $\sigma_{min}>\frac{\left. \frac{d\beta\left( \rho\right)}{d\rho} \right\vert_{\rho=0}}{\left. \frac{d\beta\left( \rho\right)}{d\rho} \right\vert_{\rho=0}-\beta(0)}$ | S53 |
| --- | --- |

### Imperfect quarantining

The fitness gradient for model A is,

| $\mathcal{F}(\rho)=\frac{-S^{*}\left( \left( -1+\eta\left( \delta-1 \right)\left( \rho-1 \right)\sigma\right)\frac{d\beta(\rho)}{d\rho}+\beta\left( \rho\right)\eta\sigma\left( \delta-1 \right) \right)}{d+\alpha+\gamma}$ | S54 |
| --- | --- |

As above, there is selection for detection avoidance when $\mathcal{F}\left( 0 \right)>0$:

| $\mathcal{F}\left( 0 \right)=\frac{-S^{*}\left( \left( -1+\eta\left( \delta-1 \right)\left( 0-1 \right)\sigma\right)\left. \frac{d\beta\left( \rho\right)}{d\rho} \right\vert_{\rho=0}+\beta\left( 0 \right)\eta\sigma\left( \delta-1 \right) \right)}{d+\alpha+\gamma}=\frac{-S^{*}}{d+\alpha+\gamma}\left( \left( -1-\eta\left( \delta-1 \right)\sigma\right)\left. \frac{d\beta\left( \rho\right)}{d\rho} \right\vert_{\rho=0}-\beta\left( 0 \right)\eta(\delta-1)\sigma\right)>0$ | S55 |
| --- | --- |

Hence,

| $\left( -1-\eta\left( \delta-1 \right)\sigma\right)\left. \frac{d\beta\left( \rho\right)}{d\rho} \right\vert_{\rho=0}-\beta\left( 0 \right)\eta(\delta-1)\sigma<0$ | S56 |
| --- | --- |

We can rearrange S56 in terms of $\sigma$ to find the minimum testing probability needed for detection avoidance to evolve:

| $\sigma_{min}>\frac{-\left. \frac{d\beta\left( \rho\right)}{d\rho} \right\vert_{\rho=0}}{\eta\left( \delta-1 \right)\left( \left. \frac{d\beta\left( \rho\right)}{d\rho} \right\vert_{\rho=0}-\beta\left( 0 \right) \right)}$ | S57 |
| --- | --- |

## Model B

### Perfect compliance and quarantining

The fitness gradient for Model B when there is perfect compliance and quarantining is,

| $\mathcal{F}\left( \rho\right)=\frac{d\beta\left( \rho\right)}{d\rho}\left( \frac{\zeta\left( 1-\rho\right)+d+\alpha+\gamma}{\beta\left( \rho\right)} \right)+\zeta$ | S58 |
| --- | --- |

There is selection for detection avoidance when $\mathcal{F}\left( 0 \right)>0:$

| $\mathcal{F}\left( 0 \right)=\left. \frac{d\beta\left( \rho\right)}{d\rho} \right\vert_{\rho=0}\left( \frac{\zeta+d+\alpha+\gamma}{\beta\left( 0 \right)} \right)+\zeta>0$ | S59 |
| --- | --- |

We can rearrange this to find the minimum testing rate needed for detection avoidance to evolve:

| $\zeta>\frac{-\left. \frac{d\beta\left( \rho\right)}{d\rho} \right\vert_{\rho=0}\left( d+\alpha+\gamma\right)}{\left. \frac{d\beta\left( \rho\right)}{d\rho} \right\vert_{\rho=0}+\beta\left( 0 \right)}$ | S60 |
| --- | --- |

### Imperfect quarantining

The fitness function for the general case is

| $\mathcal{F}(\rho)=\frac{1}{\left( d+\alpha+\gamma\right)\left( \zeta\left( 1-\rho\right)+d+\alpha+\gamma\right)^{2}}\left( \left( \left( \zeta\left( 1-\rho\right)+d+\alpha+\gamma\right)\left( -\left( 1+\left( \delta-1 \right)\eta\right)\left( \rho-1 \right)\zeta+d+\alpha+\gamma\right)\frac{d\beta\left( \rho\right)}{d\rho}-\eta\zeta\beta(\rho)\left( \delta-1 \right)\left( d+\alpha+\gamma\right) \right)S^{*} \right)$ | S61 |
| --- | --- |

Here we will write $\tilde{d}=d+\alpha+\gamma$ for brevity, hence

| $\mathcal{F}(\rho)=\frac{1}{\tilde{d}\left( \zeta\left( 1-\rho\right)+\tilde{d} \right)^{2}}\left( \left( \left( \zeta\left( 1-\rho\right)+\tilde{d} \right)\left( -\left( 1+\left( \delta-1 \right)\eta\right)\left( \rho-1 \right)\zeta+\tilde{d} \right)\frac{d\beta\left( \rho\right)}{d\rho}-\eta\zeta\beta(\rho)\left( \delta-1 \right)\tilde{d} \right)S^{*} \right)$ | S62 |
| --- | --- |

Selection favours detection avoidance when $\mathcal{F}\left( 0 \right)>0$:

| $\mathcal{F}\left( 0 \right)=\frac{1}{\tilde{d}\left( \zeta+\tilde{d} \right)^{2}}\left( \left( \left( \zeta+\tilde{d} \right)\left( -\left( 1+\left( \delta-1 \right)\eta\right)\left( -1 \right)\zeta+\tilde{d} \right)\left. \frac{d\beta\left( \rho\right)}{d\rho} \right\vert_{\rho=0}-\eta\zeta\beta\left( 0 \right)\left( \delta-1 \right)\tilde{d} \right)S^{*} \right)>0$ | S63 |
| --- | --- |

Hence,

| $\left( \zeta+\tilde{d} \right)\left( -\left( 1+\left( \delta-1 \right)\eta\right)\left( -1 \right)\zeta+\tilde{d} \right)\left. \frac{d\beta\left( \rho\right)}{d\rho} \right\vert_{\rho=0}-\eta\zeta\beta\left( 0 \right)\left( \delta-1 \right)\tilde{d}>0$ | S64 |
| --- | --- |

This can be rearranged to obtain a quadratic in $\zeta$, which can be solved to determine upper and lower bounds on testing rates for detection avoidance to evolve.

## Model C

### Perfect compliance and quarantining

The fitness gradient for Model C when there is perfect compliance and quarantining is,

| $\mathcal{F}(\rho)=\frac{\left( \rho\zeta+\left( d+\alpha+\gamma\right)) \beta^{'}\left( \rho\right)+\zeta\beta\left( \rho\right) \right)S^{*}}{(d+\alpha+\gamma)(\zeta+d+\alpha+\gamma)}$ | S65 |
| --- | --- |

There is selection for detection avoidance when $\mathcal{F}\left( 0 \right)>0,$ hence

| $\mathcal{F}\left( 0 \right)=(d+\alpha+\gamma)\left. \frac{d\beta\left( \rho\right)}{d\rho} \right\vert_{\rho=0}+\zeta\beta(0)>0$ | S66 |
| --- | --- |

We can rearrange this to find the minimum testing rate needed for detection avoidance to evolve:

| $\zeta_{min}>\frac{-(d+\alpha+\gamma)\left. \frac{d\beta\left( \rho\right)}{d\rho} \right\vert_{\rho=0}}{\beta(0)}$ | S67 |
| --- | --- |

### Imperfect quarantining

The fitness function for the general case is

| $\mathcal{F}(\rho)=\frac{\left( \left( -1+\left( -1+\rho\right)\left( \delta-1 \right)\eta\right)\zeta-\left( d+\alpha+\gamma\right)) \beta^{'}\left( \rho\right)+\eta\zeta\beta\left( \rho\right)\left( \delta-1 \right) \right)S^{*}}{(d+\alpha+\gamma)(\zeta+d+\alpha+\gamma)}$ | S68 |
| --- | --- |

Here we will write $\tilde{d}=d+\alpha+\gamma$ for brevity, hence

| $\mathcal{F}(\rho)=\frac{\left( \left( -1+\left( -1+\rho\right)\left( \delta-1 \right)\eta\right)\zeta-\tilde{d}) \beta^{'}\left( \rho\right)+\eta\zeta\beta\left( \rho\right)\left( \delta-1 \right) \right)S^{*}}{\tilde{d}(\zeta+\tilde{d})}$ | S69 |
| --- | --- |

Selection favours detection avoidance when $\mathcal{F}\left( 0 \right)>0$:

| $\mathcal{F}\left( 0 \right)=\frac{\left( \left( -1-\left( \delta-1 \right)\eta\right)\zeta-\tilde{d}) \left. \frac{d\beta\left( \rho\right)}{d\rho} \right\vert_{\rho=0}+\eta\zeta\beta\left( 0 \right)\left( \delta-1 \right) \right)S^{*}}{\tilde{d}(\zeta+\tilde{d})}>0$ | S70 |
| --- | --- |

Hence,

| $\zeta_{min}>\frac{\tilde{d}\left. \frac{d\beta\left( \rho\right)}{d\rho} \right\vert_{\rho=0}}{\left. \frac{d\beta\left( \rho\right)}{d\rho} \right\vert_{\rho=0}\left( -1-\delta\eta+\eta\right)+\eta\beta\left( 0 \right)\left( \delta-1 \right)}$ | S71 |
| --- | --- |

# Supplementary figures


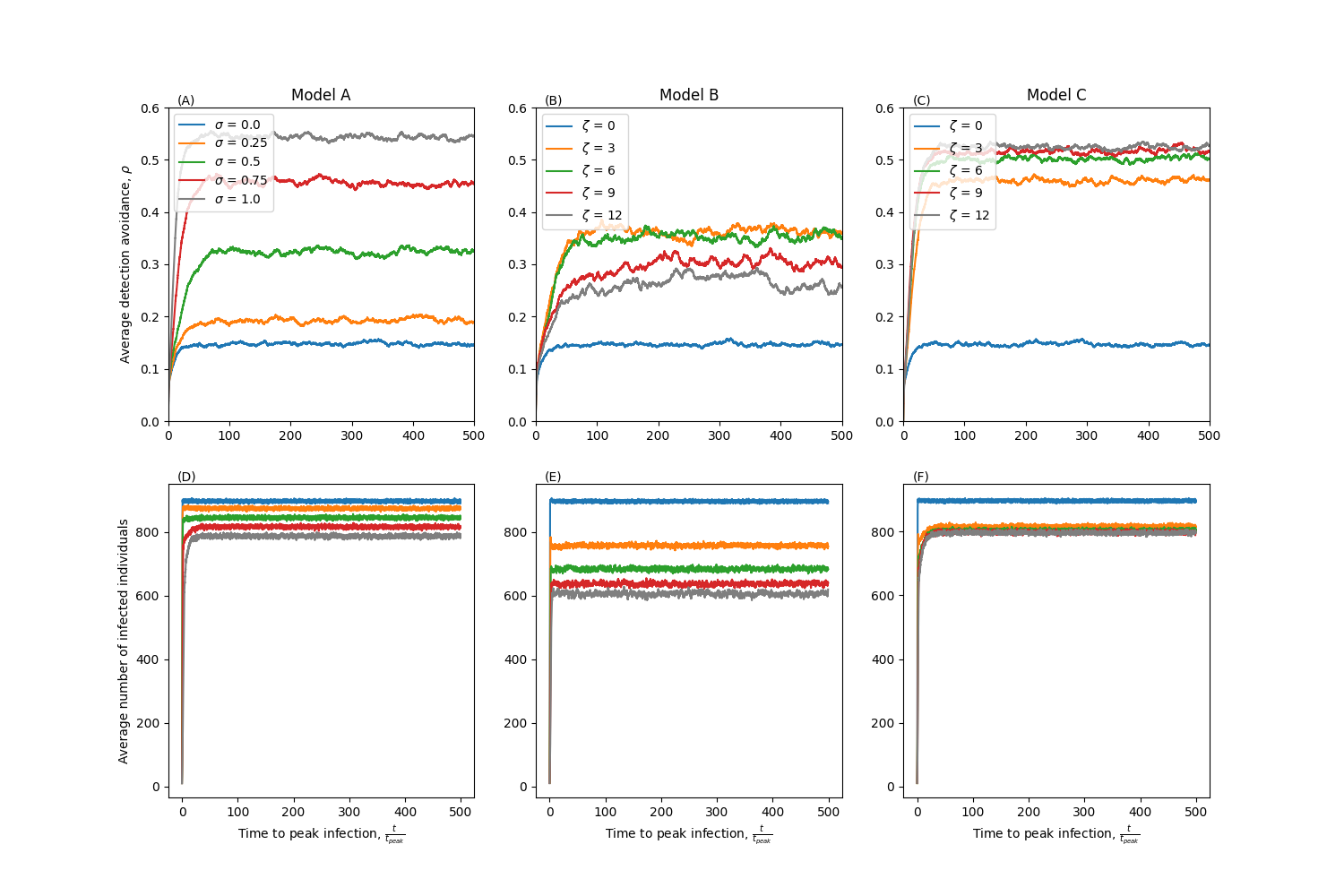


Figure S1: Stochastic simulations of dynamics for Models A-C (A-C). The first row of figures show the average detection avoidance. The second row of figures show the average number of infected individuals. Parameters as in Table 1 except 𝛿=0.1, 𝜂=0.90, 𝛽=0.01.

**
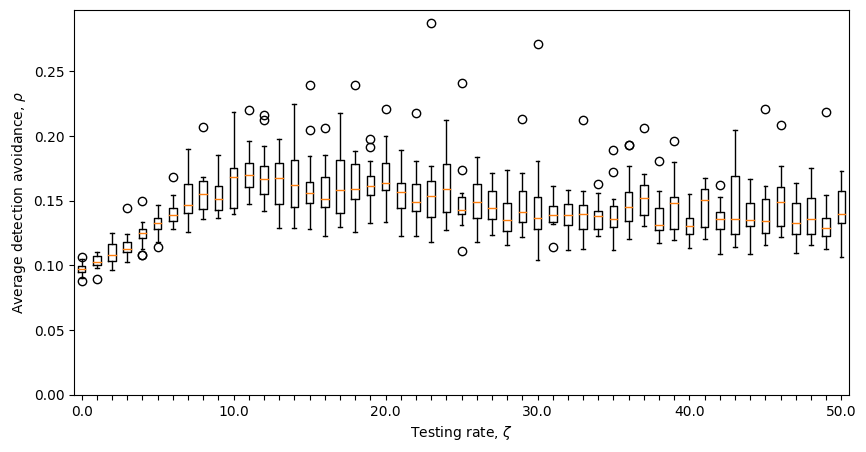
**

Figure S2: Box plot of average detection avoidance $\rho$at peak number of infected individuals for Model B. Parameters as in Table 1 except $\delta=0.1, \eta=0.9, \beta=0.00001$.


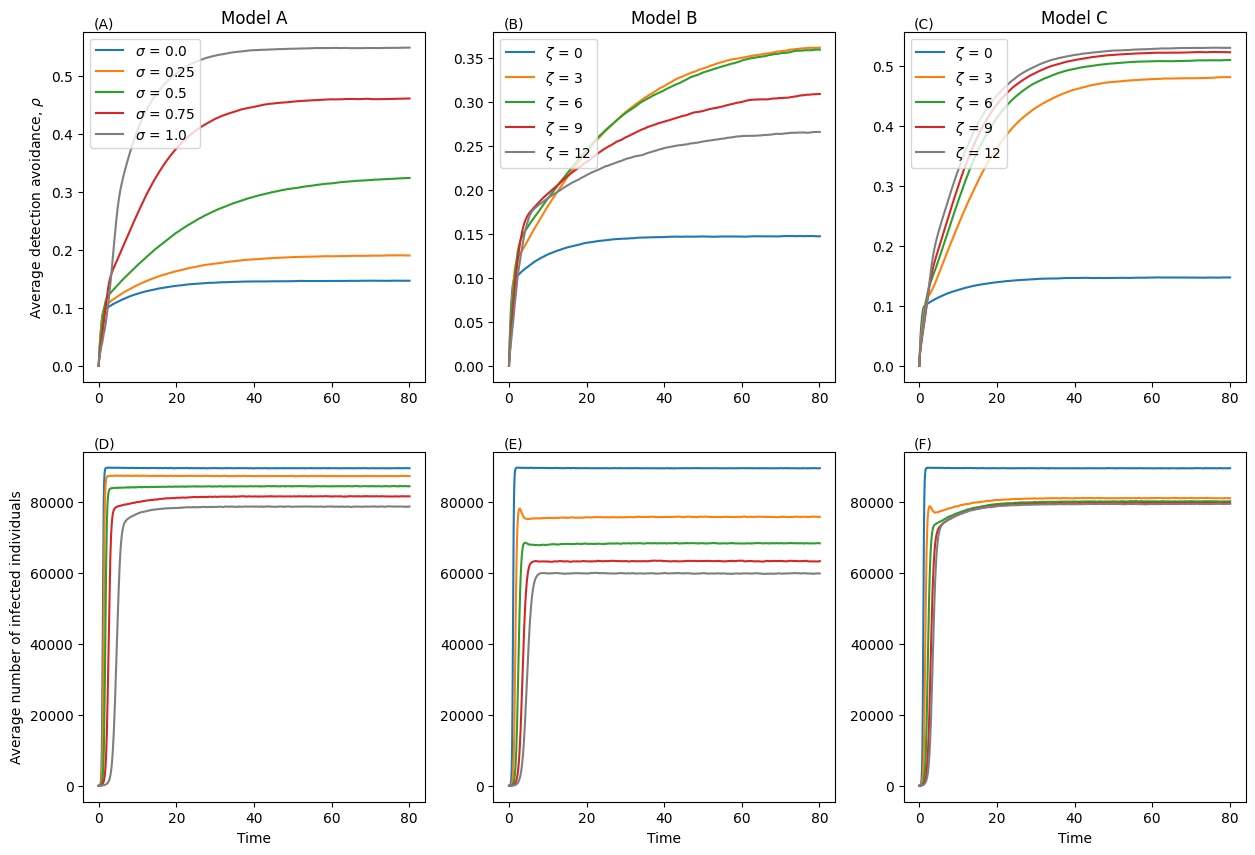


Figure S3: Stochastic simulations of dynamics for Models A-C (A-C). The first row of figures show the average detection avoidance. The second row of figures show the average number of infected individuals. Parameters as in Table 1 except 𝛿=0.1, 𝜂=0.90,$\beta=0.0001, N=100,000.$
